# Supplementary material for: Proteomics of colorectal tumors identifies the role of CAVIN1 in tumor relapse
Source: Mol Syst Biol. 2025 Apr 23;21(7):776–806. doi: 10.1038/s44320-025-00102-8 (PMC12222889; doi:10.1038/s44320-025-00102-8)
Supplement: Supplementary file 1 — Appendix [file 44320_2025_102_MOESM1_ESM.pdf]

Appendix for:

Proteomics of colorectal tumors identifies the role of CAVIN1 in tumor relapse

Ana Martinez-Val[1,4,5*], Leander Van der Hoeven[1], Dorte B. Bekker-Jensen[1,65], Margarita Melnikova Jørgensen, Jesper Nors[2,3], Giulia Franciosa[1*], Claus L. Andersen[2,3*], Jesper B. Bramsen[2,3*], Jesper V. Olsen[1*]

[1] Novo Nordisk Foundation Center for Protein Research, Department of Cellular and Molecular Medicine, Faculty of Health and Medical Sciences, University of Copenhagen, Copenhagen, DENMARK
[2] Department of Molecular Medicine, Aarhus University Hospital, Aarhus, DENMARK
[3]Department of Clinical Medicine, Aarhus University Hospital, Aarhus, DENMARK
[4] Current address: Laboratory of Cardiovascular Proteomics. Centro Nacional de Investigaciones Cardiovasculares (CNIC), Madrid 28029, SPAIN.
[5] Current address: CIBER de Enfermedades Cardiovasculares (CIBERCV), Madrid, SPAIN.
[6] Current address: Evosep Biosystems, Odense, DENMARK

Table of contents:

- Appendix Figure S1: page 2
- Appendix Figure S2 page 3
- Appendix Figure S3: page 4
- Appendix Figure S4: page 5
- Appendix Figure S5: page 6
- Appendix Figure S6: page 7
- Appendix Figure S7: page 8
- Appendix Figure S8: page 9

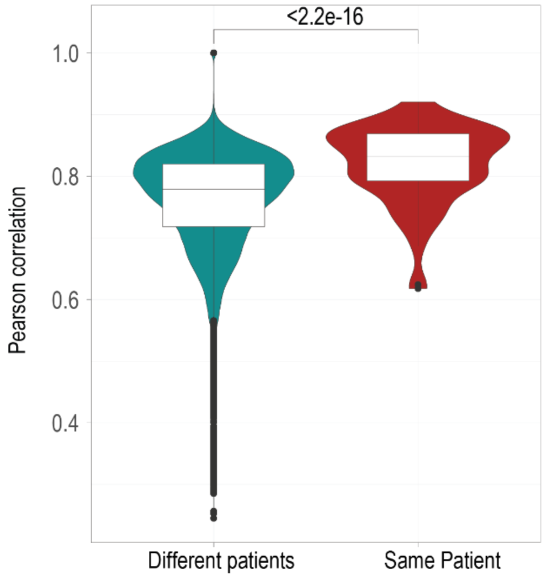

Appendix Figure S1: Intra-patient variability. Boxplot showing the Pearson correlation between the proteomics profiles of samples obtained from different patients (n=126,622), and samples obtained from the same patient (n=114). P-value was calculated for a two-sample t-test statistical analysis.

A

All (stage II and III)	1	2	3	4	Total
**YES**	19	25	16	15	75
**NO**	55	111	45	70	281
**Total**	74	136	61	85	356
**expected**	15.590	28.652	12.851	17.907	
**pval**	0.175	0.200	0.179	0.234	
**direction**	enriched	de-enriched	enriched	de-enriched	

B

All (stage II and III) - CMS	CMS1	CMS2	CMS3	CMS4	Total
**YES**	6	24	16	23	69
**NO**	65	88	41	68	262
**Total**	71	112	57	91	331
**expected**	14.801	23.347	11.882	18.970	
**pval**	**0.002**	0.479	0.100	0.143	
**direction**	de-enriched	enriched	enriched	enriched	

C

Stage III	1	2	3	4	Total
**YES (III)**	16	16	7	5	44
**NO (III)**	25	49	11	33	118
**Total**	41	65	18	38	162
**expected**	11.136	17.654	4.889	10.321	
**pval**	**0.040**	0.341	0.181	**0.019**	
**direction**	enriched	de-enriched	enriched	de-enriched	

D

Stage III	CMS1	CMS2	CMS3	CMS4	Total
**YES (III)**	2	10	13	16	41
**NO (III)**	28	30	16	35	109
**Total**	30	40	29	51	150
**expected**	8.200	10.933	7.927	13.940	
**pval**	**0.002**	0.435	0.019	0.271	
**direction**	de-enriched	de-enriched	enriched	enriched	

Appendix Figure S2. Results of hypergeometric test (two-sided) indicating the statistical over- or under-representation or patients that suffered relapse in each assigned molecular subtype. (A) Results for proteomics subtypes using all patients independently of tumour stage. (B) Results for transcriptomics subtypes using all patients independently of tumour stage. (C) Results for proteomics subtypes using only stage III patients. (D) Results for transcriptomics subtypes using only stage III patients.

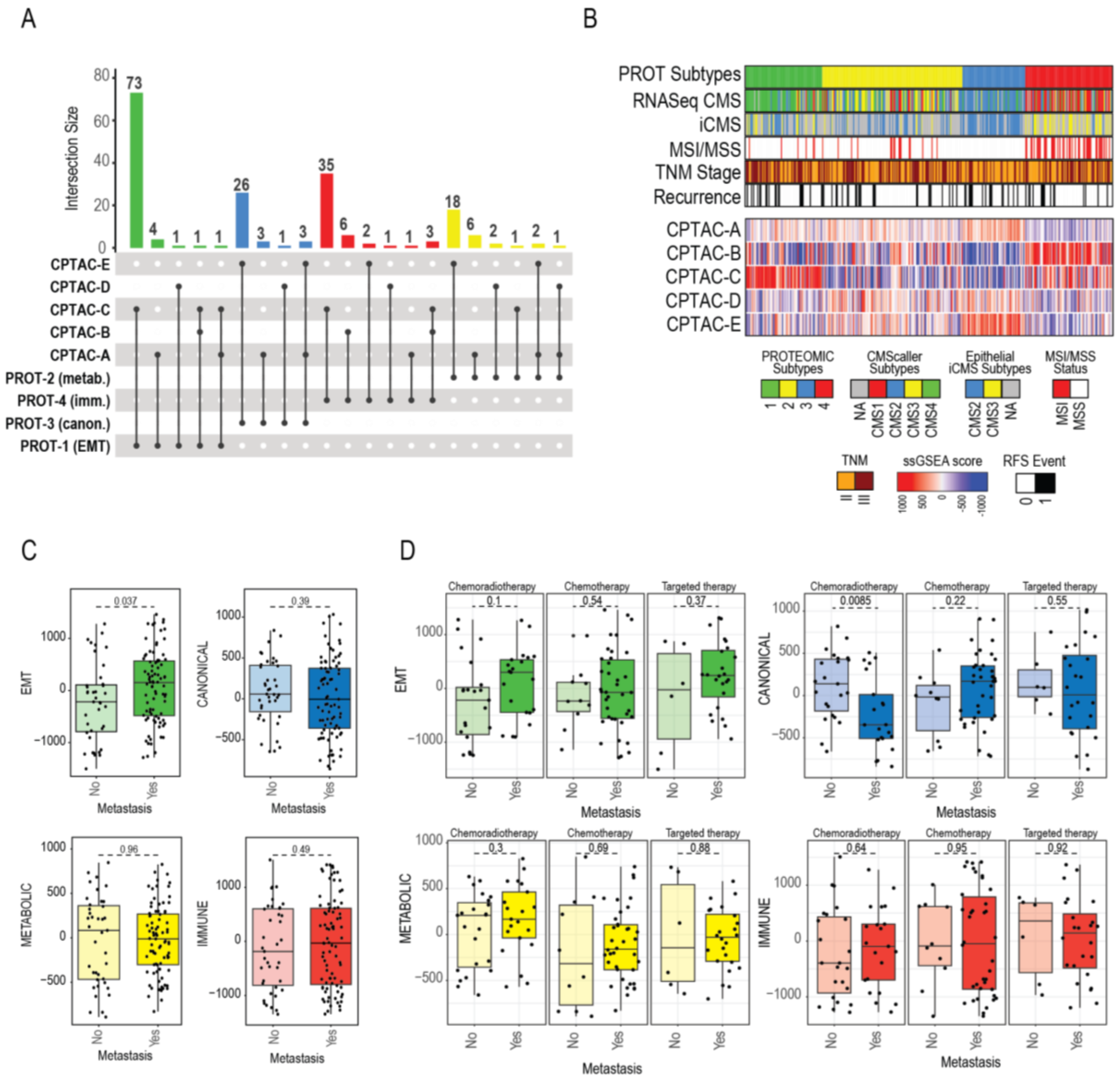

Appendix Figure S3. Comparison with published datasets. (A) Upset plot reflecting the intersection (proteins in common) between the proteomics-based molecular signatures defined in this paper (PROT-1, PROT-2, PROT-3 and PROT-4) and those defined by Zhang et al (PMID: 25043054). (B) Heatmap showing the patient ssGSEA results using Zhang et al up-regulated protein signatures (PMID: 25043054) on the present study samples (1st cohort) grouped based on the proteomics subtype defined in this work. (C) Boxplot of ssGSEA scores obtained using the four proteomics signatures described in this work (PROT1: EMT, PROT2: METABOLIC, PROT3: CANONICAL, PROT4: IMMUNE) on the Li, Wang et al data (PMID: 38086380), grouped as a function of metastasis. On top, p-value from a two-sample t-test analysis. (D) Boxplot of ssGSEA scores obtained using the four proteomics signatures described in this work (PROT1: EMT, PROT2: METABOLIC, PROT3: CANONICAL, PROT4: IMMUNE) on the Li, Wang et al data, grouped as a function of metastasis treatment received. On top, p-value from a two-sample t-test analysis.

A

B

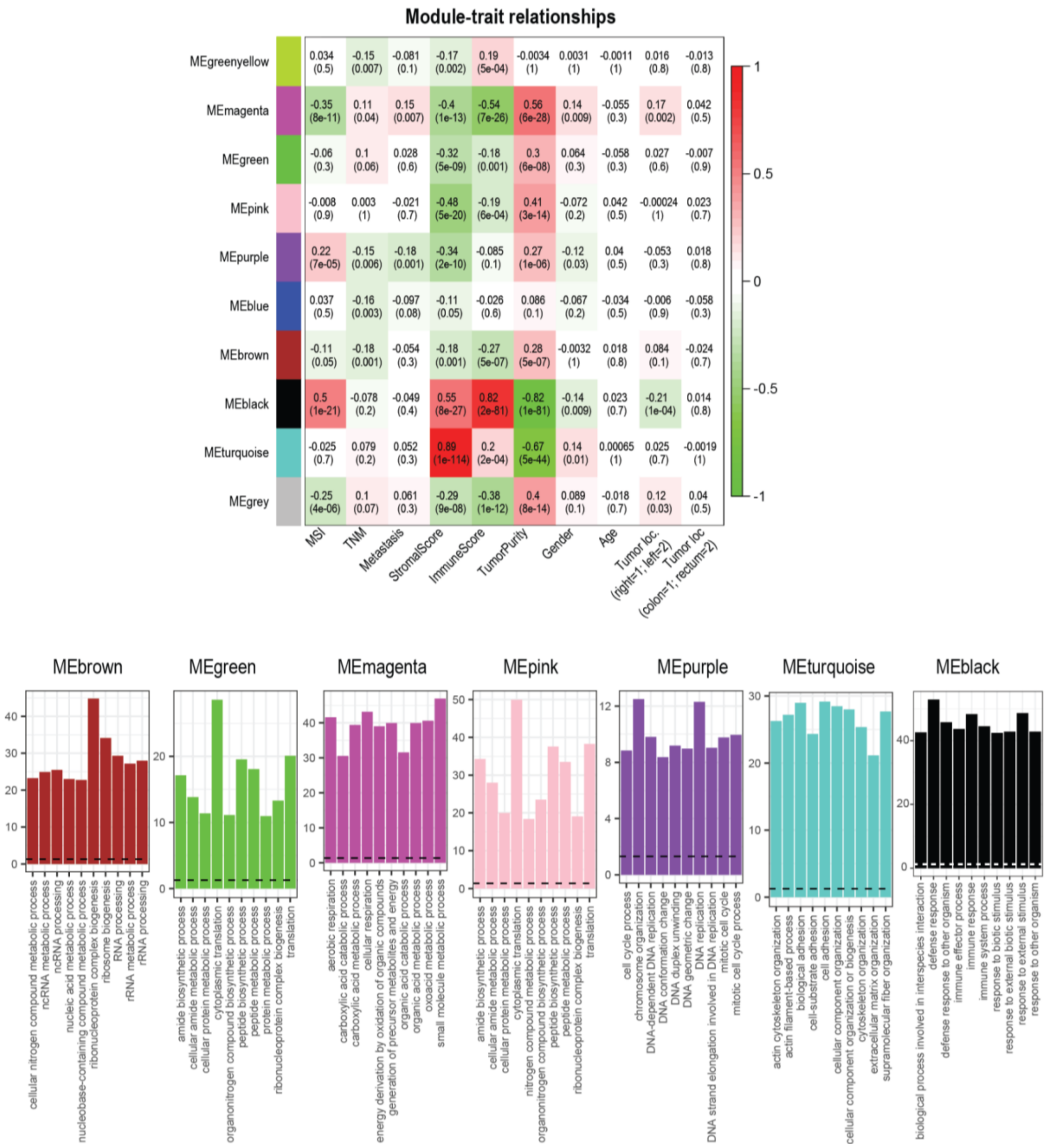

Appendix Figure S4: Weighted Gene Coexpression Network analysis of proteomics data and clinical traits. (A) Heatmap of the correlation between module eigengenes and clinical traits of colorectal cancer patients. MSS/MSI, TNM, Relapse, Gender and Tumor Location were binary values: MSS/MSI: MSS=1, MSI=2; TNM: Stages 2 and 3; Relapse: Yes= 1, No=0; Gender: Female=0, Male=1; Tumor location; left=1, right=2 and Tumor location, colon=1; rectum=2. (B) Barplots with Gene Ontology Biological Process terms overrepresented in each of the indicated modules.

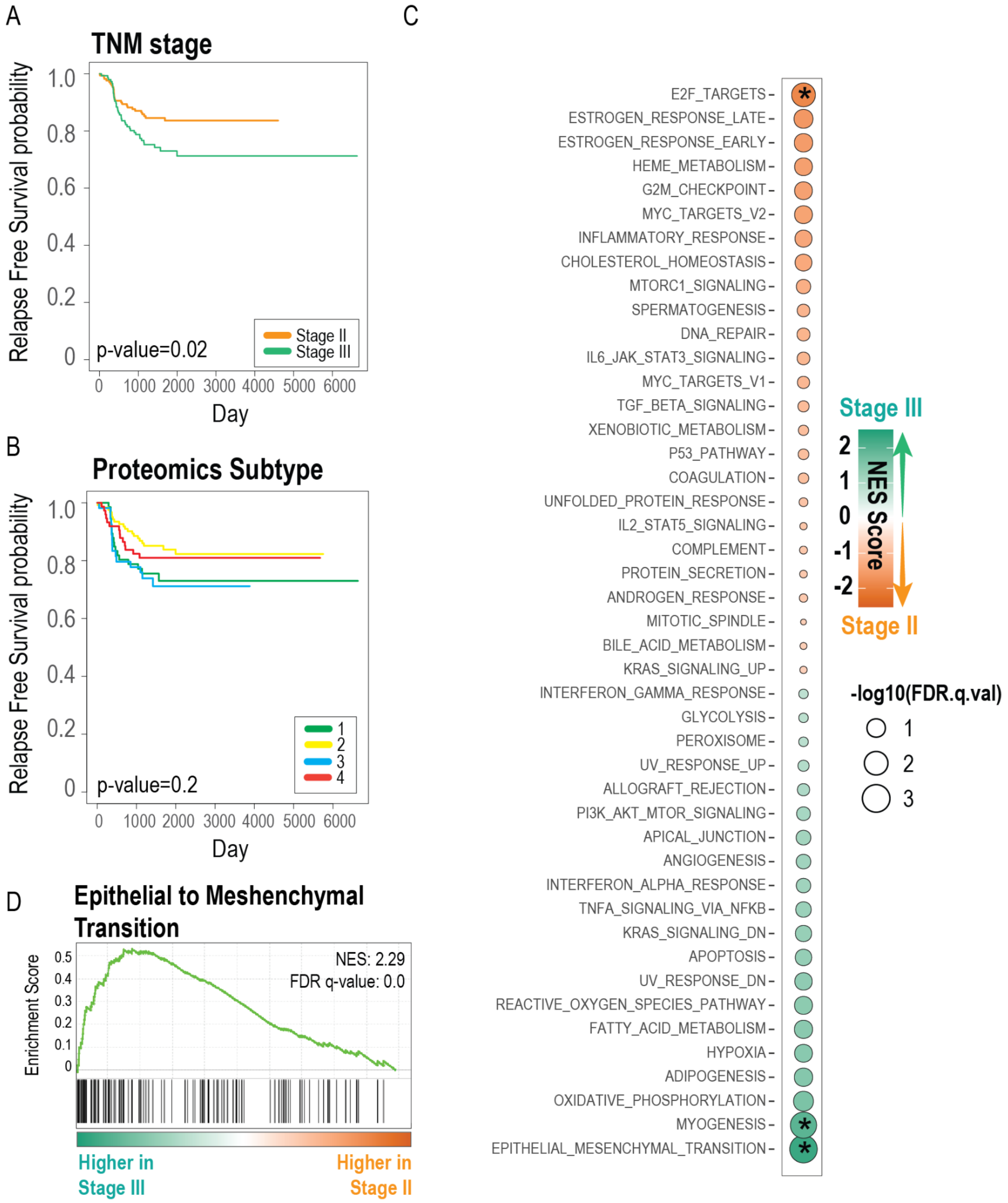

Appendix Figure S5: Evaluation of molecular pathways linked to development of colorectal cancer. (A) Kaplan-Meier curve analysis comparing relapse-free survival probabilities in patients diagnosed in stage II or in stage III.(B) Kaplan-Meier curve analysis comparing relapse-free survival probabilities in the different proteomics subtypes. (C) Gene Set Enrichment Analysis (GSEA) between patients diagnosed in Stage II and stage III using the Hallmark gene sets. Size of the dot indicates the statistical significance of the enrichment and color de normalized enrichment score. Dots with an asterisk indicate that FDR q-value <0.05. (D) GSEA plot of the “Epithelial to Mesenchymal” gene set.

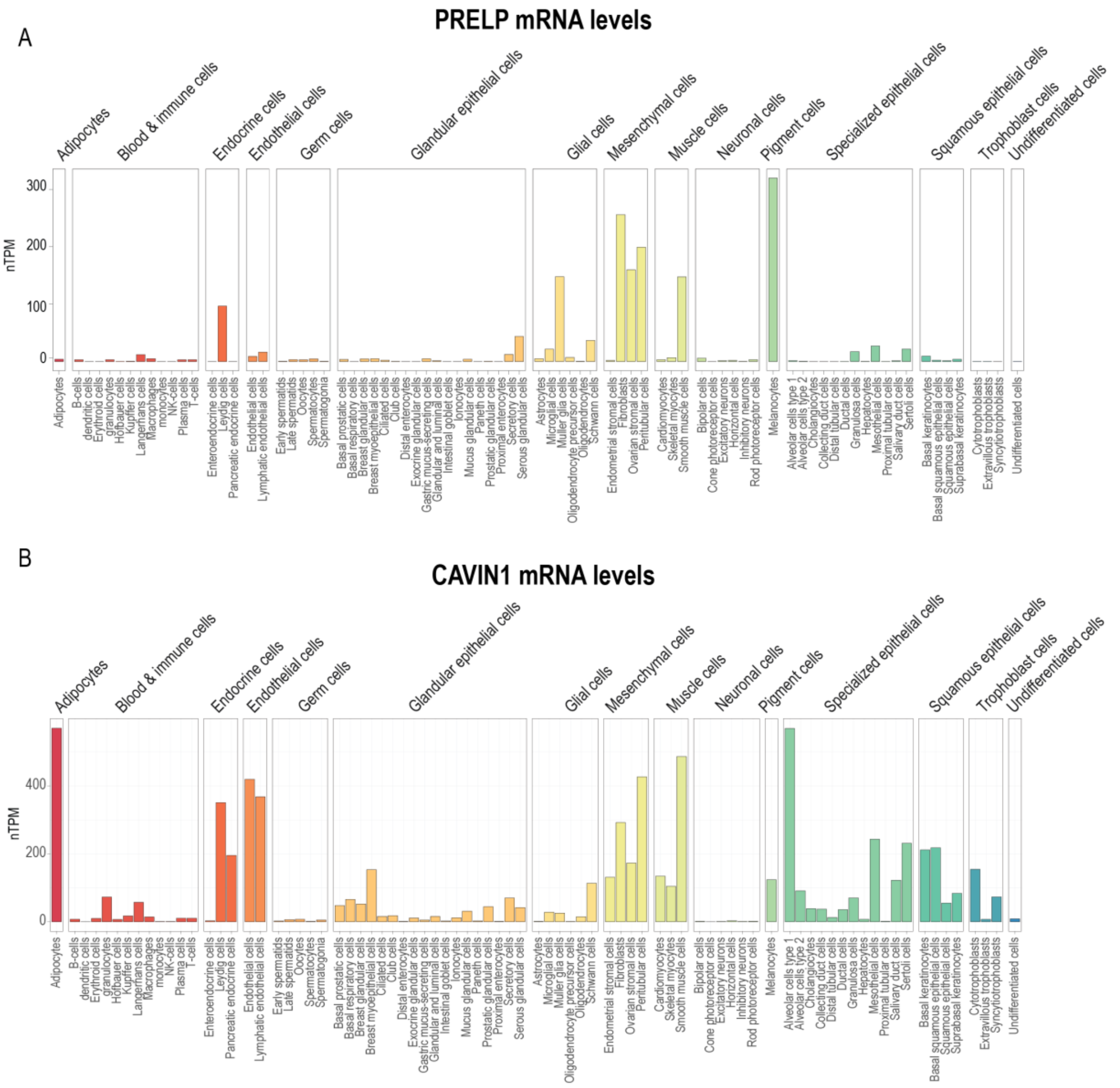

Appendix Figure S6: PRELP and CAVIN1 distribution in tissues and cell types. Transcript levels (measured as normalized transcripts per million) of PRELP (A) and CAVIN1 (B) across different cell types. Data obtained from The Human Protein Atlas web.

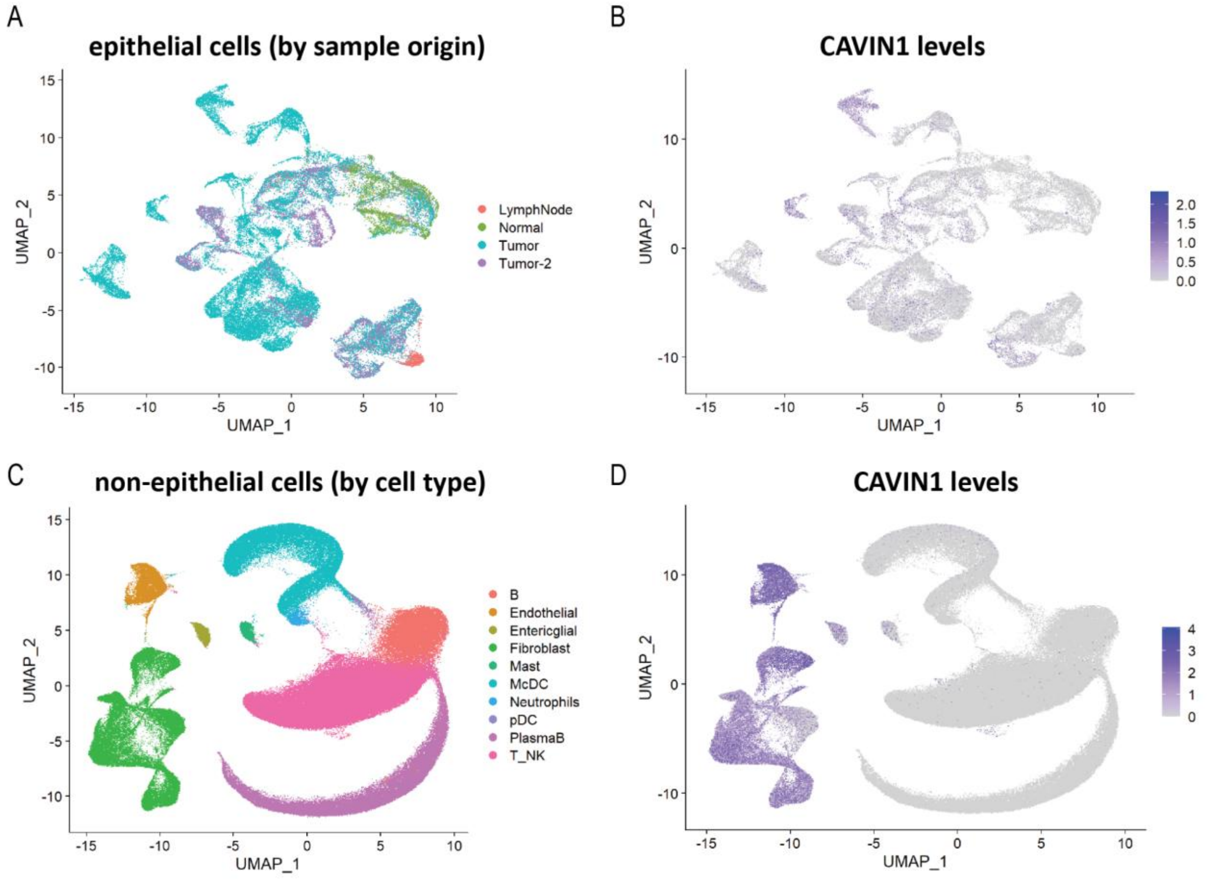

Appendix Figure S7: single cell RNASeq data colorectal cancer biopsies showing CAVIN1 levels by cell type, from Joanito et al (PMID: 35773407). (A) UMAP representation of single-cell RNASeq data from epithelial cells obtained from biopsies of colorectal cancer patients. Single cells are colored by sample of origin. (B) CAVIN1 transcript levels measured in single cells from epithelial origin in colorectal cancer samples. (C) UMAP representation of single-cell RNASeq data from non-epithelial cells obtained from biopsies of colorectal cancer patients. Single cells are colored by type pf cell. (D) CAVIN1 transcript levels measured in single cells from non-epithelial origin in colorectal cancer samples.

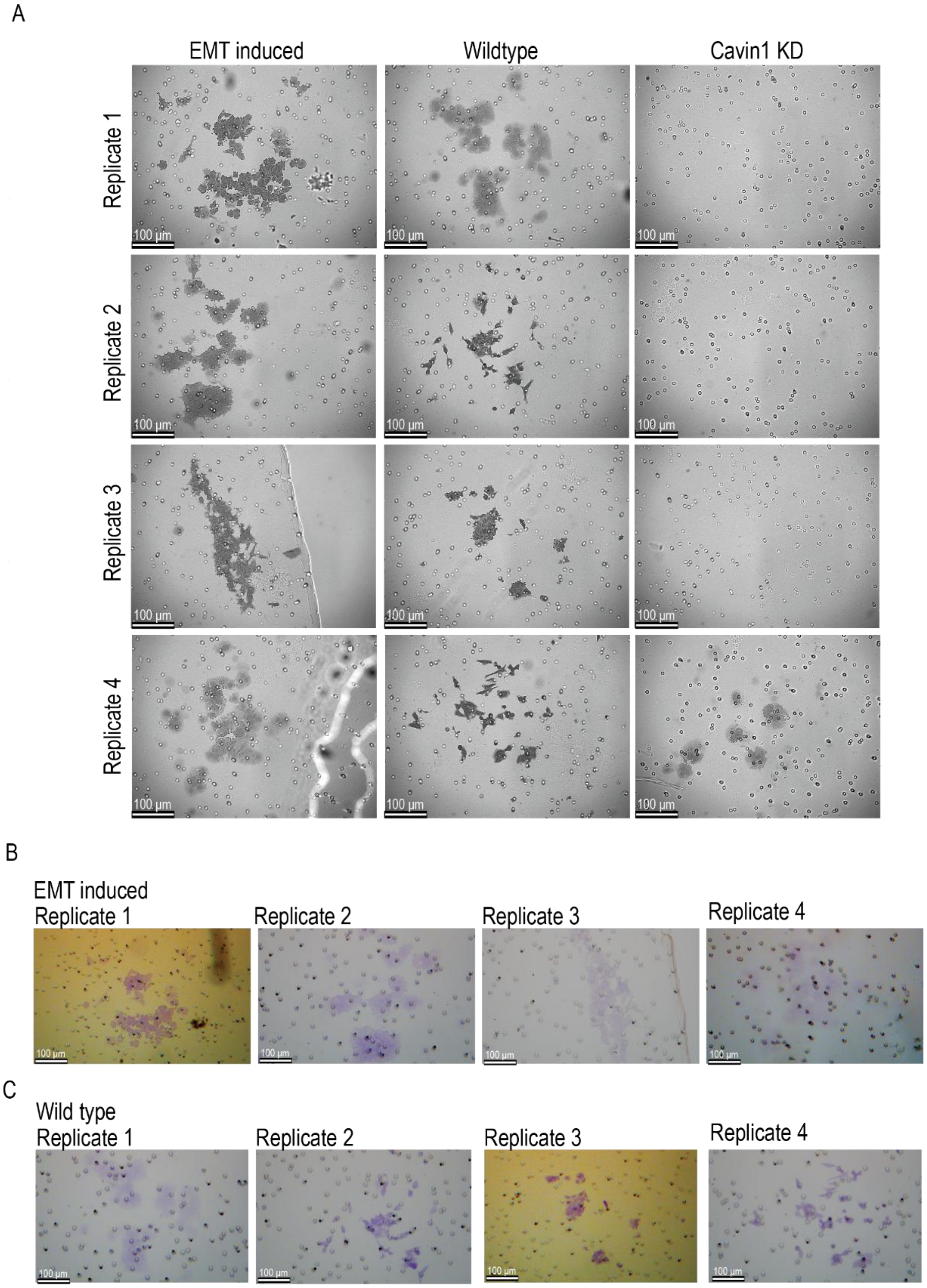

Appendix Figure S8: In vitro validation of EMT-marker CAVIN1 relation with tumor invasiveness. (A) Photographs of the bottom part of the matrigel membrane used in the Matrigel 24 well-plate 8.0 micron invasion chambers (Corning), after 24 hours of seeding one spheroid on top of them. Three conditions (four replicates each) were analyzed: upon induction of EMT with an EMT inducing cocktail, wild type and CAVIN1 knock down. (B) and (C) correspond to the same pictures shown in A, but photographed with a color camera to observe the Coomassie blue staining. This photographs are also shown in Figure 4G.
