# Supplementary figures and images for: Proteomics of colorectal tumors identifies the role of CAVIN1 in tumor relapse
Source: Mol Syst Biol. 2025 Apr 23;21(7):776–806. doi: 10.1038/s44320-025-00102-8 (PMC12222889; doi:10.1038/s44320-025-00102-8)

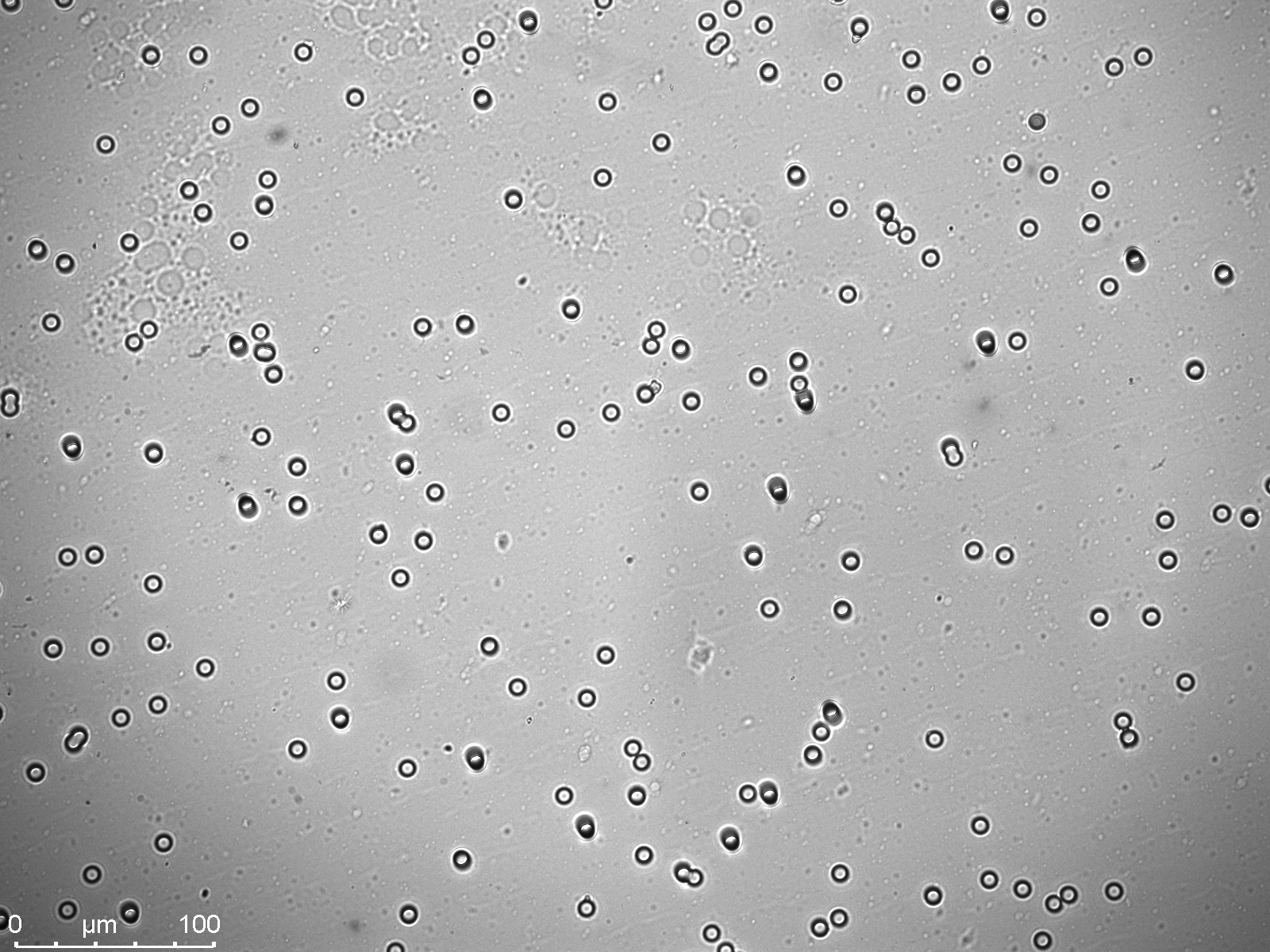

Supplement: Supplementary file 10 — Source data Fig. 4 [file 44320_2025_102_MOESM10_ESM.zip › Figure 4G/HT29_KD_Rep1.tif]

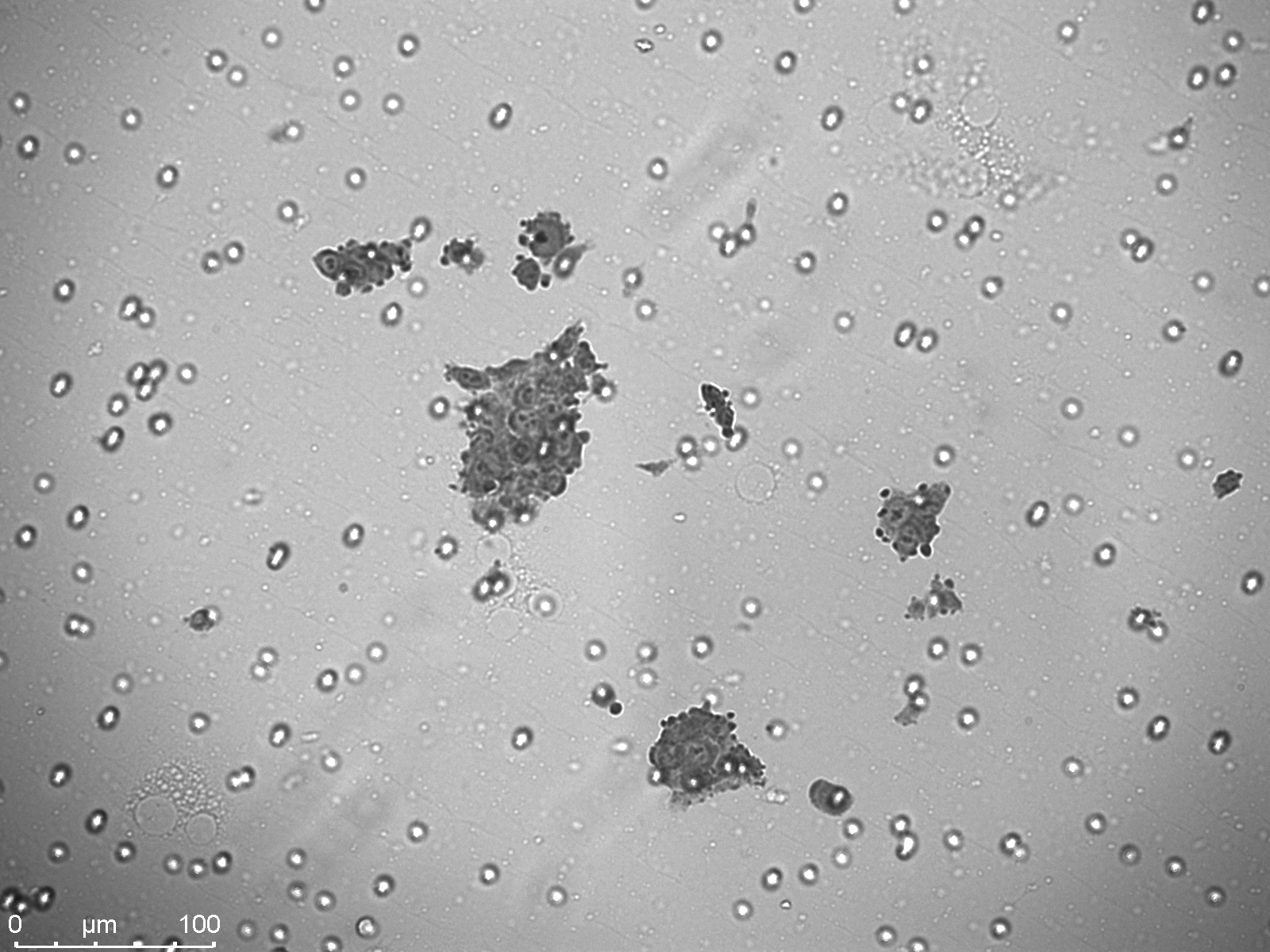

Supplement: Supplementary file 10 — Source data Fig. 4 [file 44320_2025_102_MOESM10_ESM.zip › Figure 4G/HT29_Ctrl_Rep3.tif]

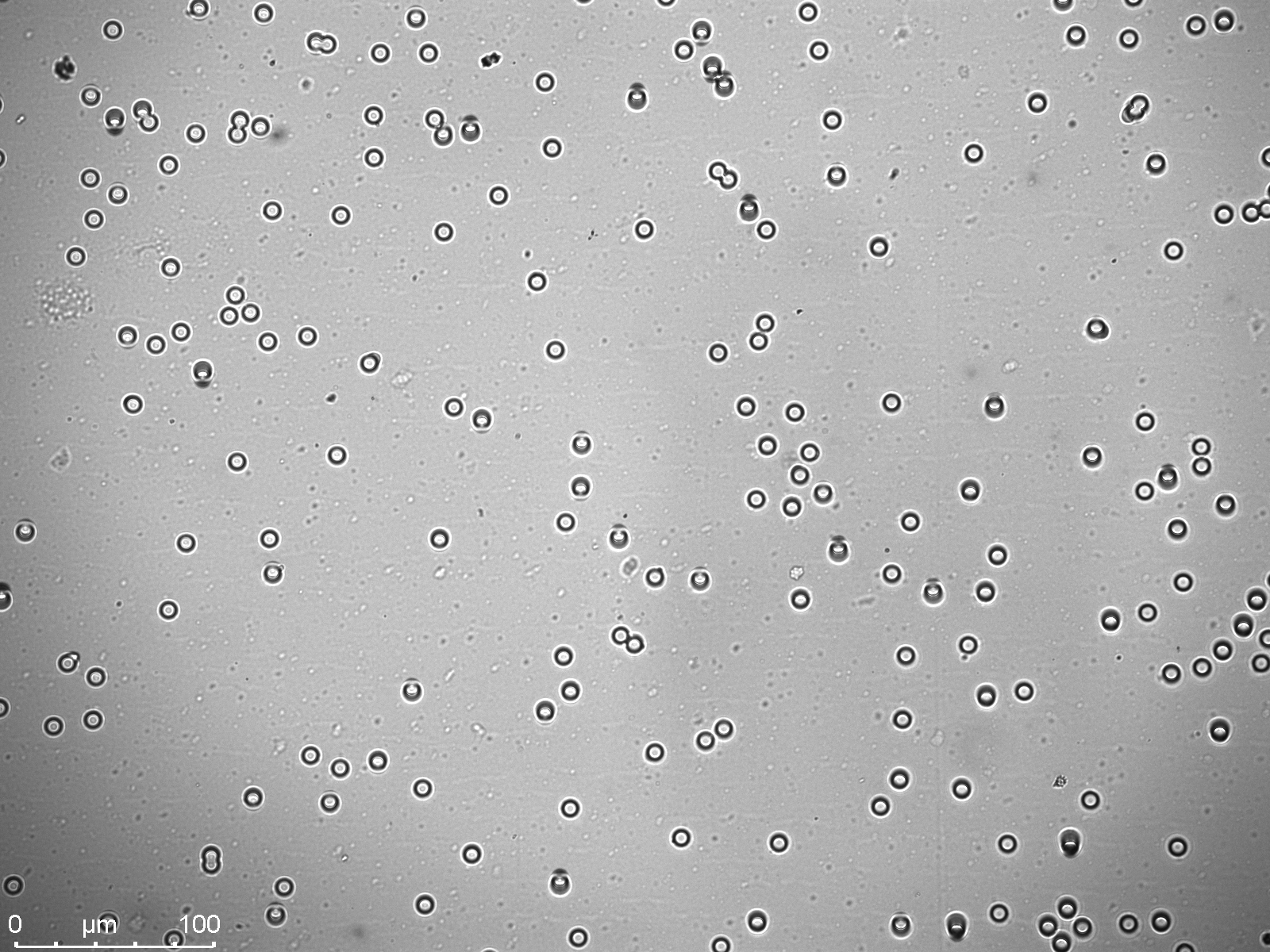

Supplement: Supplementary file 10 — Source data Fig. 4 [file 44320_2025_102_MOESM10_ESM.zip › Figure 4G/HT29_KD_Rep2.tif]

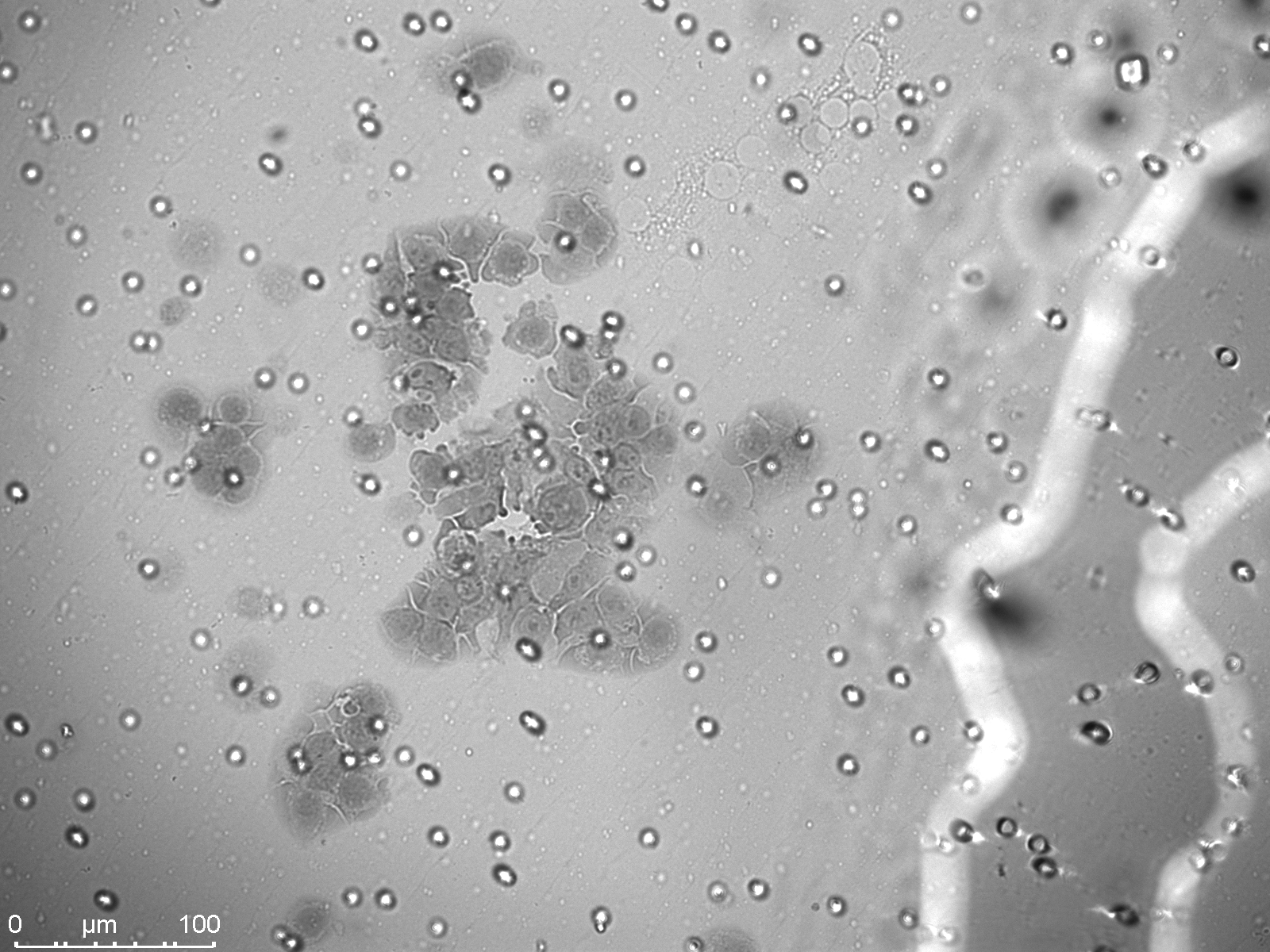

Supplement: Supplementary file 10 — Source data Fig. 4 [file 44320_2025_102_MOESM10_ESM.zip › Figure 4G/HT29_EMT_Rep4.tif]

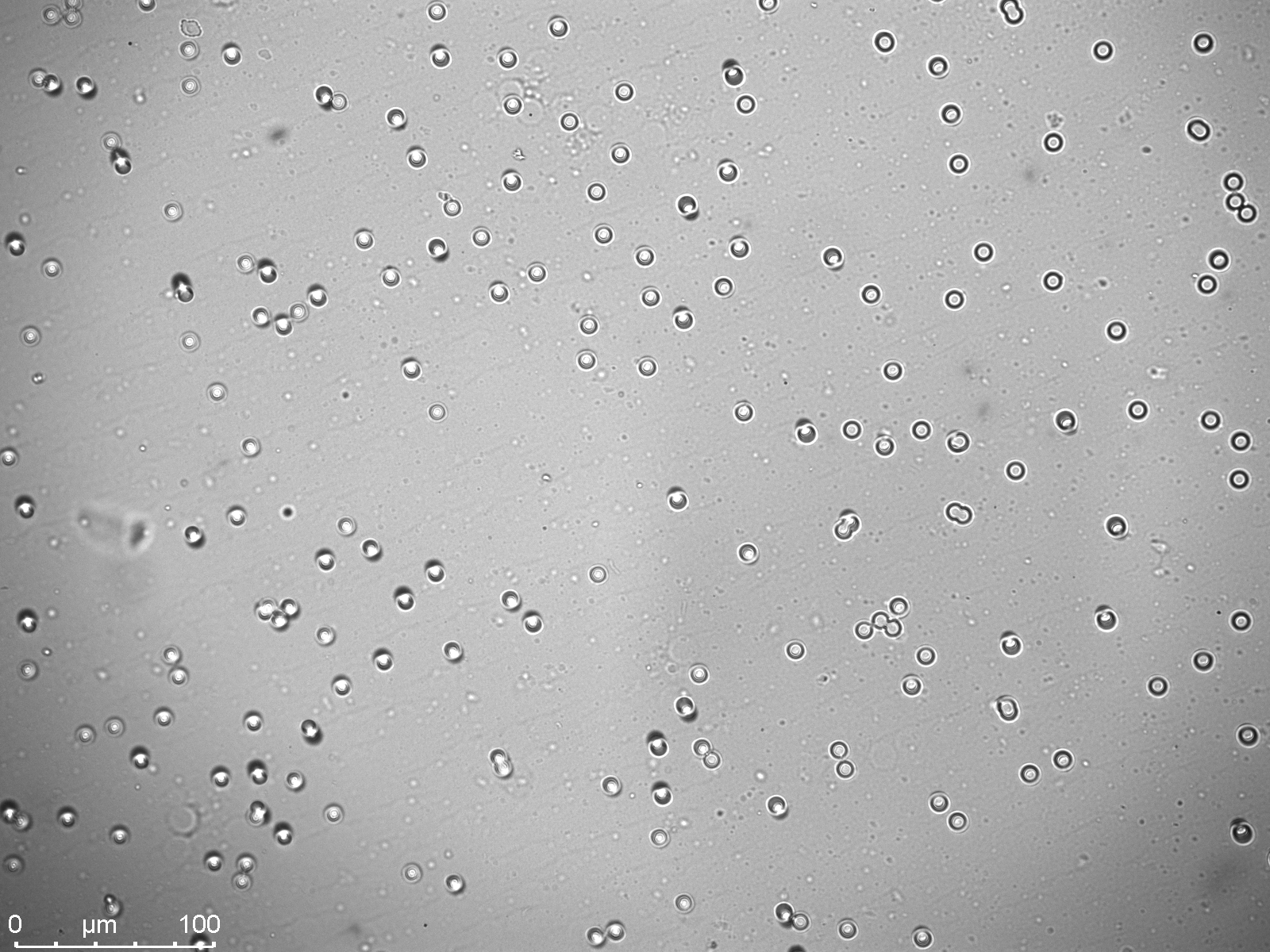

Supplement: Supplementary file 10 — Source data Fig. 4 [file 44320_2025_102_MOESM10_ESM.zip › Figure 4G/HT29_KD_Rep3.tif]

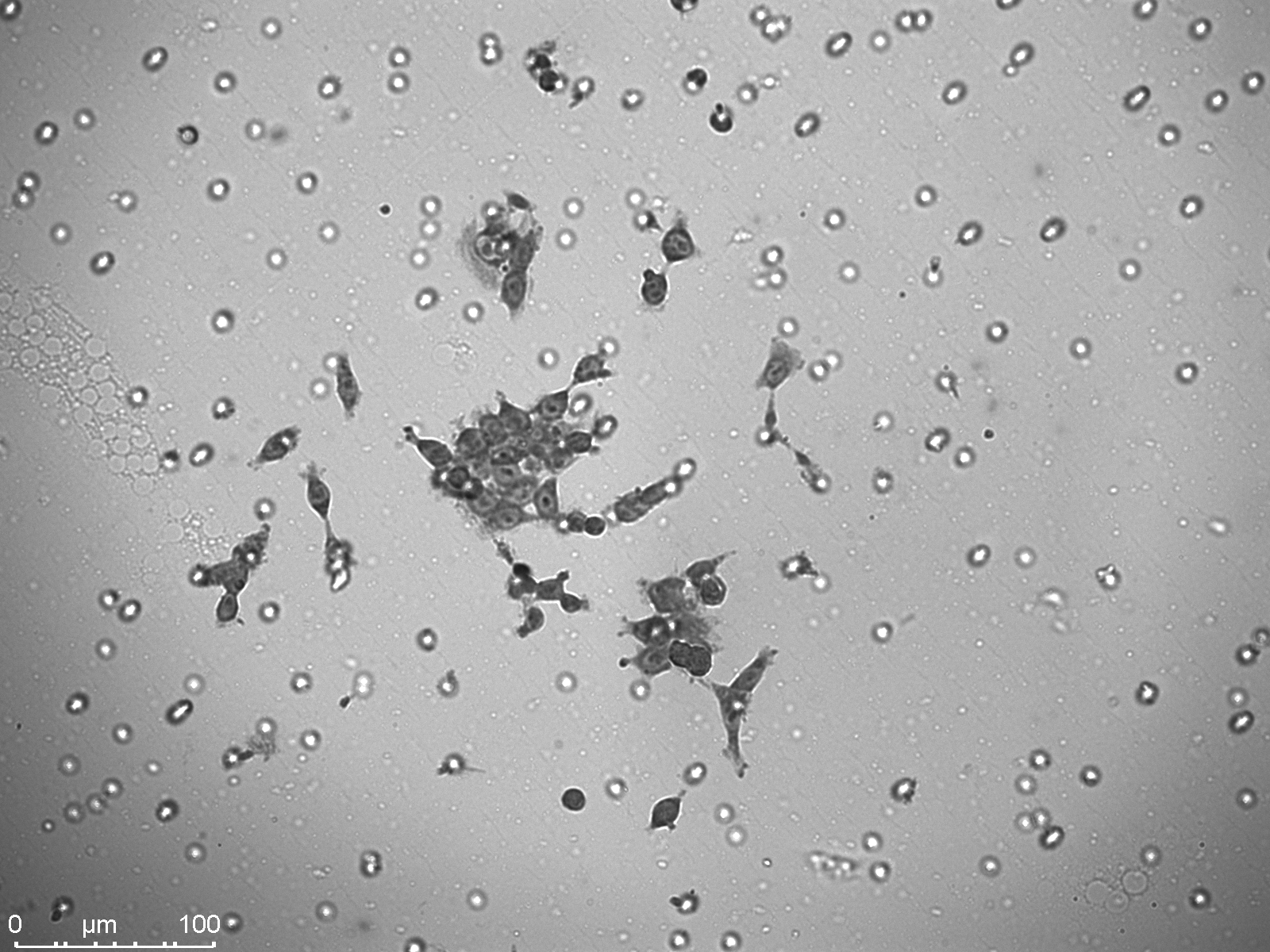

Supplement: Supplementary file 10 — Source data Fig. 4 [file 44320_2025_102_MOESM10_ESM.zip › Figure 4G/HT29_Ctrl_Rep2.tif]

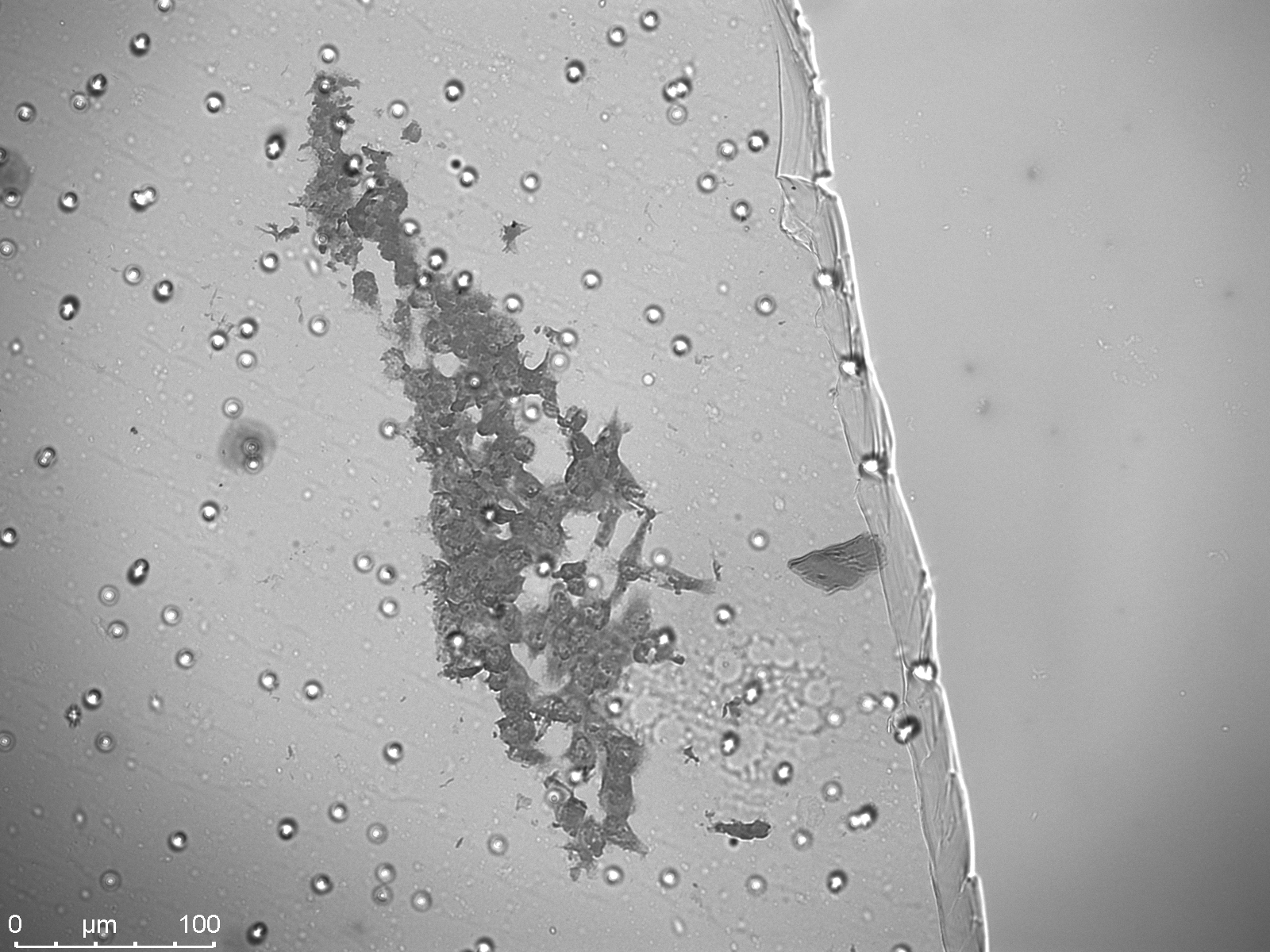

Supplement: Supplementary file 10 — Source data Fig. 4 [file 44320_2025_102_MOESM10_ESM.zip › Figure 4G/HT29_EMT_Rep3.tif]

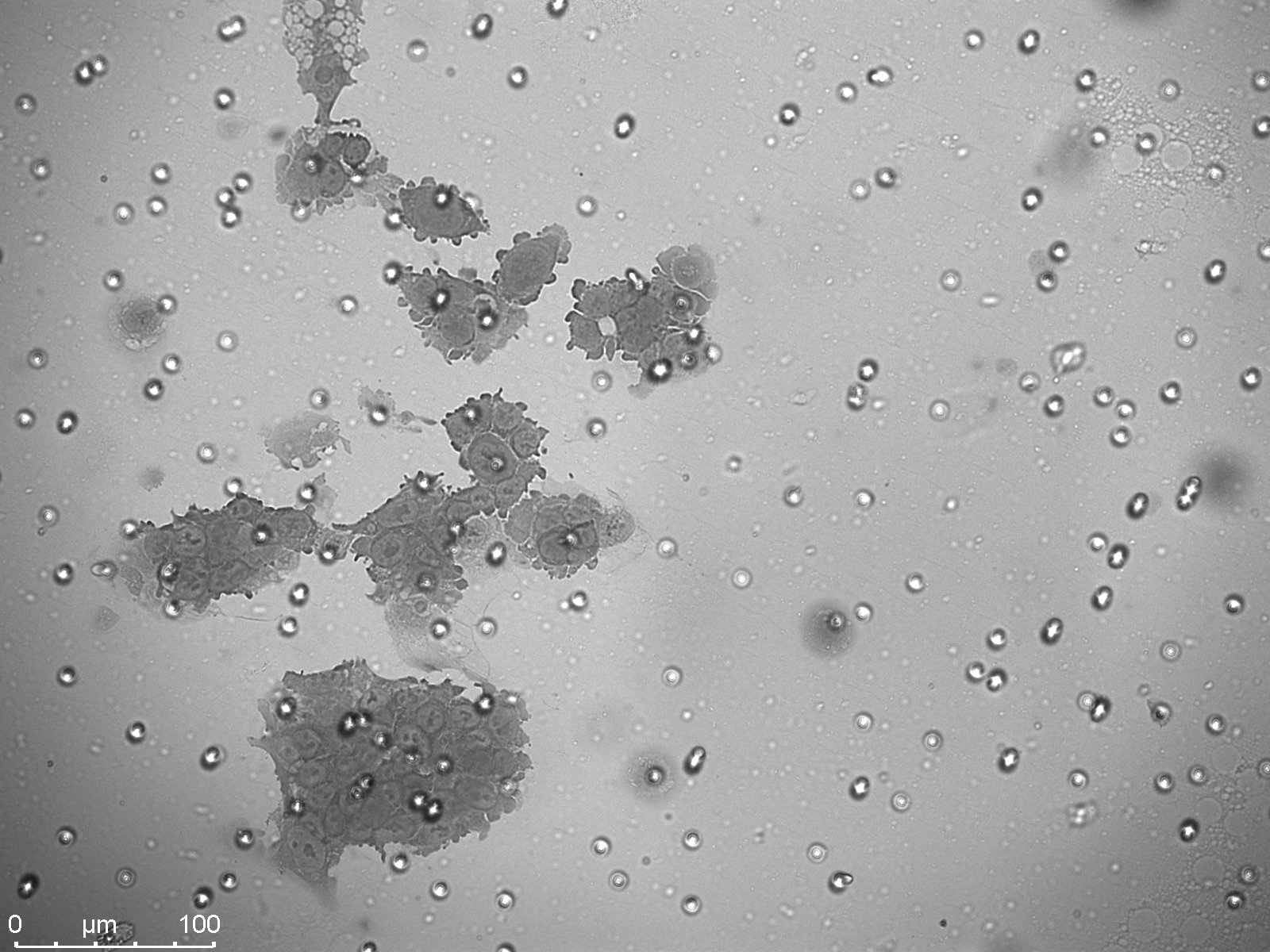

Supplement: Supplementary file 10 — Source data Fig. 4 [file 44320_2025_102_MOESM10_ESM.zip › Figure 4G/HT29_EMT_Rep2.tif]

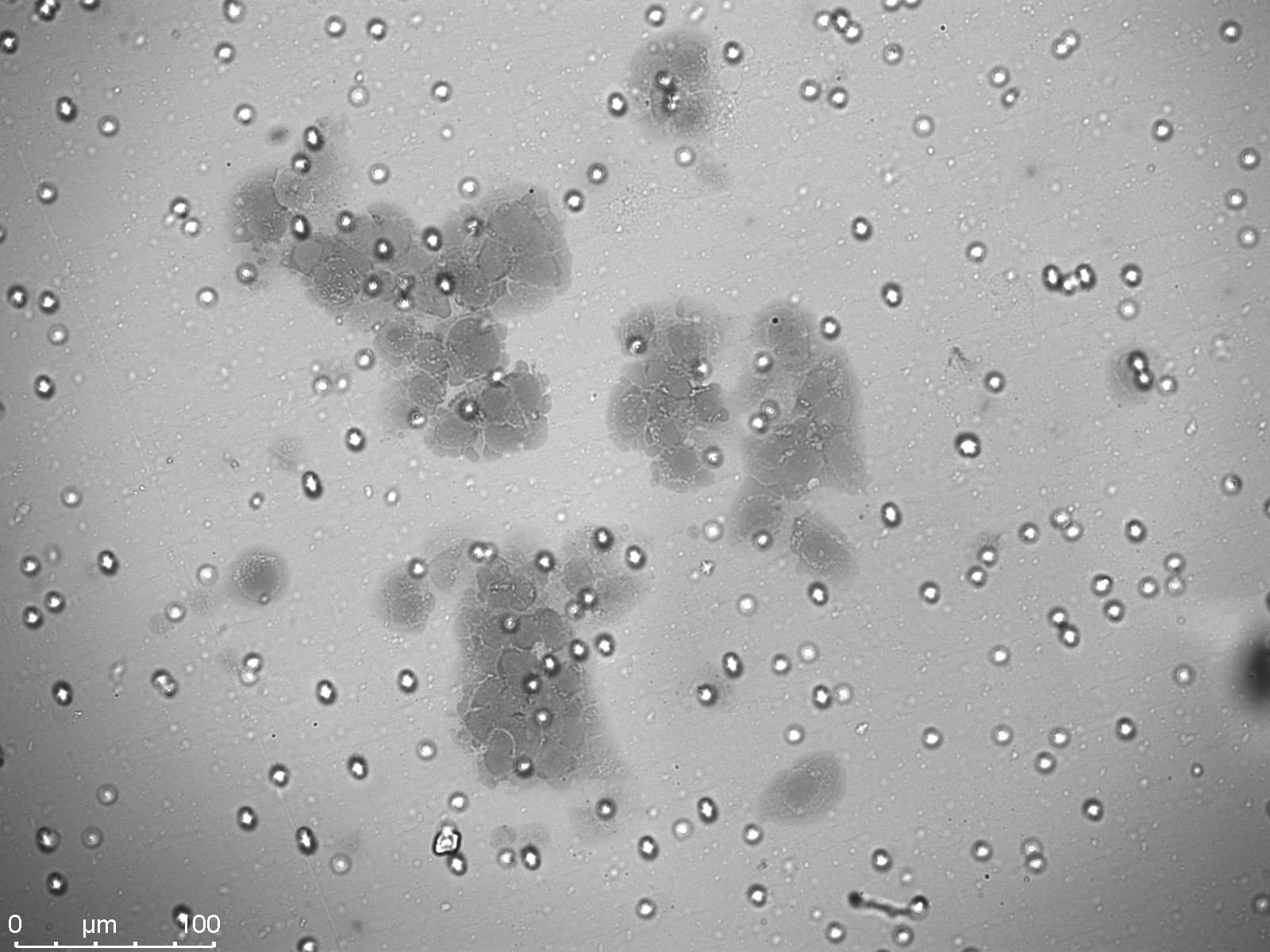

Supplement: Supplementary file 10 — Source data Fig. 4 [file 44320_2025_102_MOESM10_ESM.zip › Figure 4G/HT29_Ctrl_Rep1.tif]

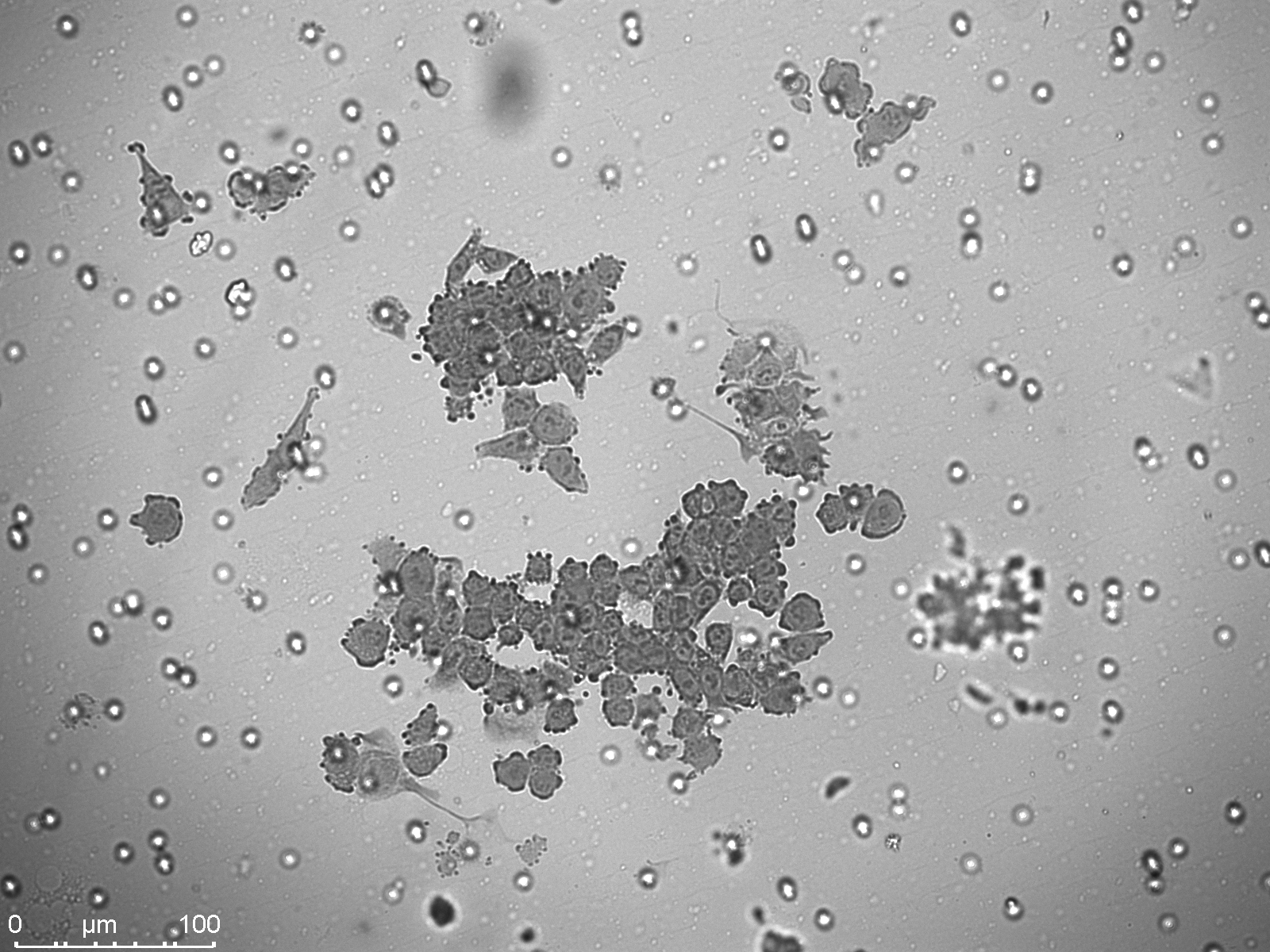

Supplement: Supplementary file 10 — Source data Fig. 4 [file 44320_2025_102_MOESM10_ESM.zip › Figure 4G/HT29_EMT_Rep1.tif]

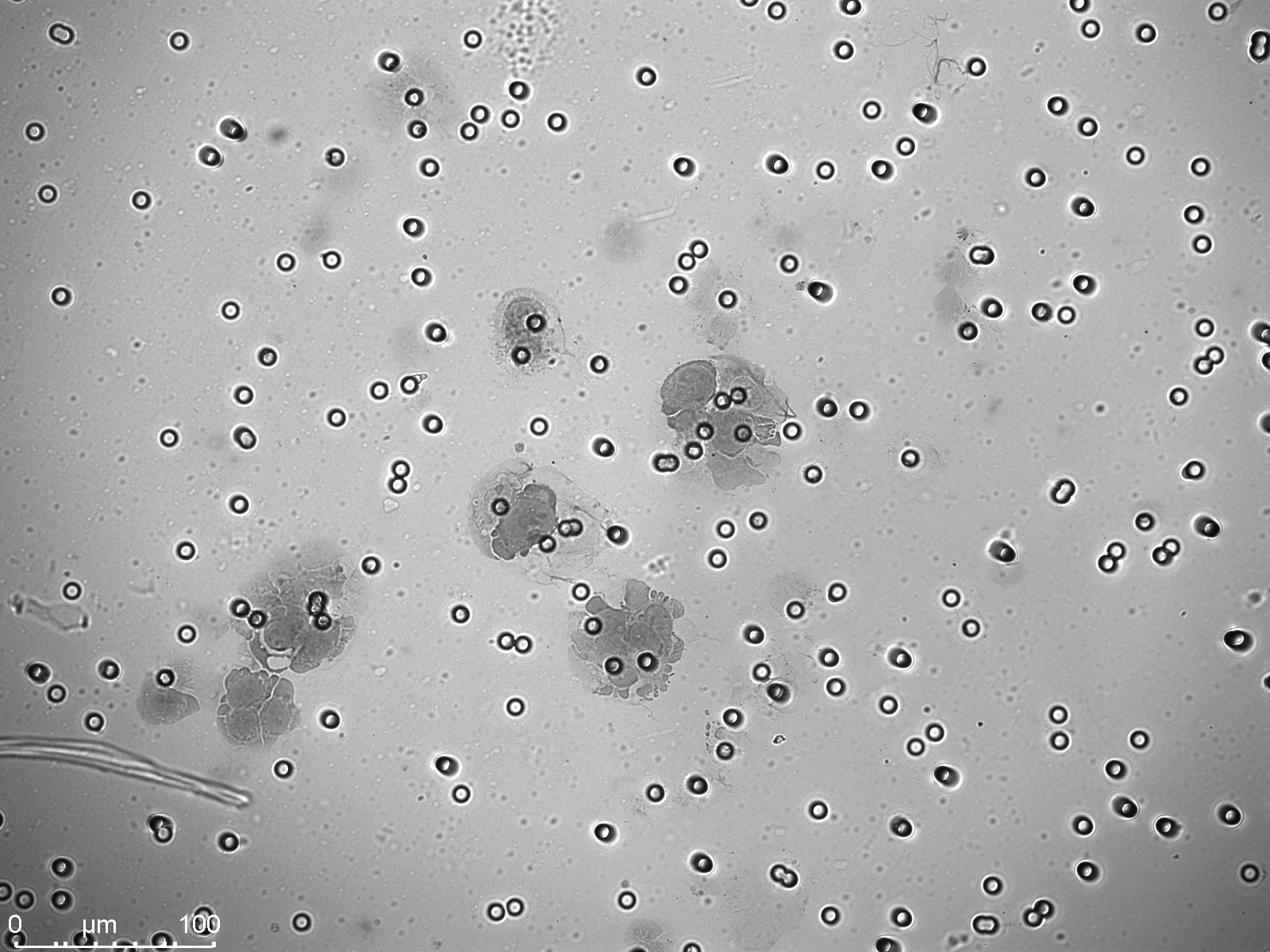

Supplement: Supplementary file 10 — Source data Fig. 4 [file 44320_2025_102_MOESM10_ESM.zip › Figure 4G/HT29_KD_Rep4.tif]

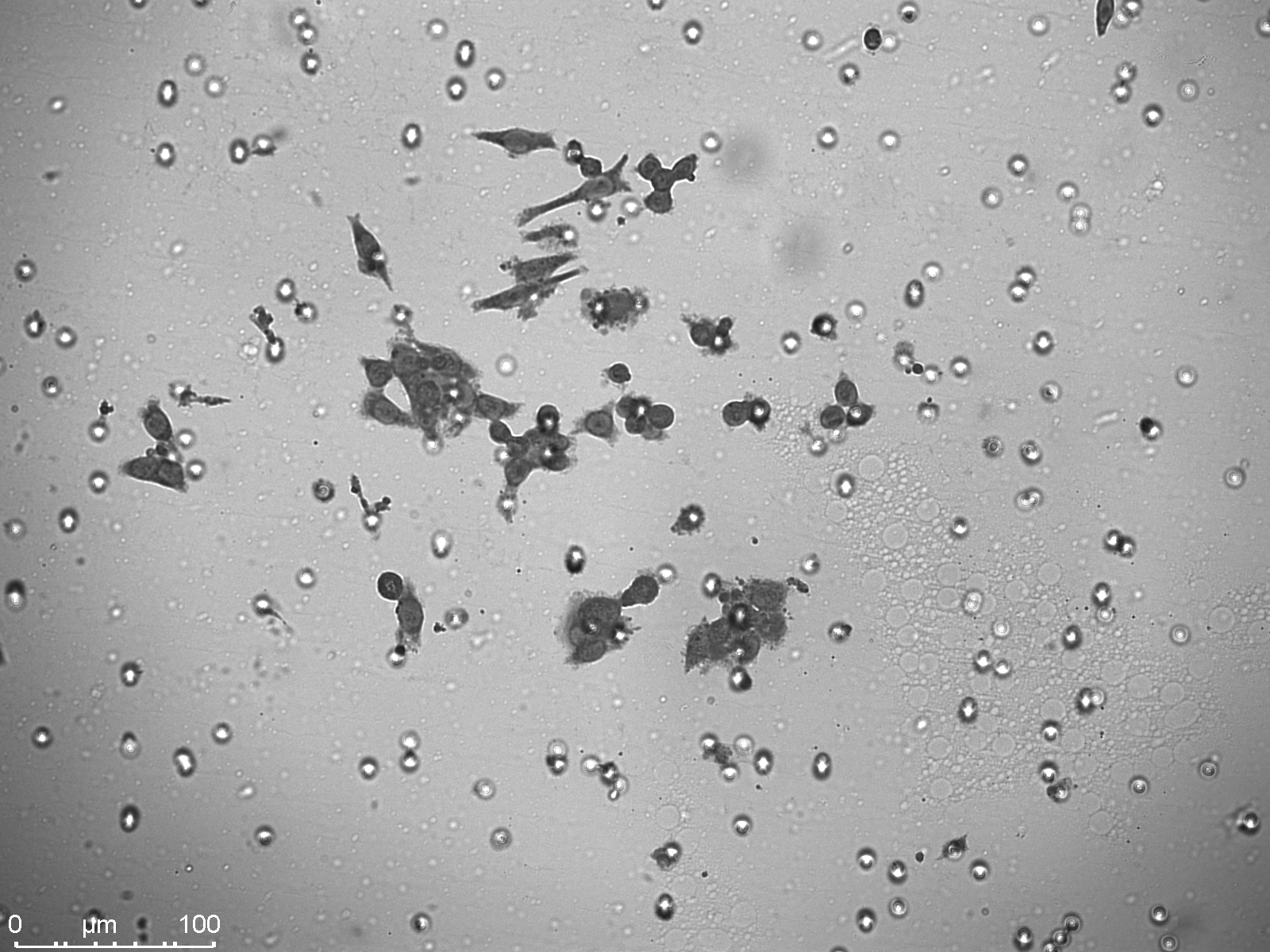

Supplement: Supplementary file 10 — Source data Fig. 4 [file 44320_2025_102_MOESM10_ESM.zip › Figure 4G/HT29_Ctrl_Rep4.tif]

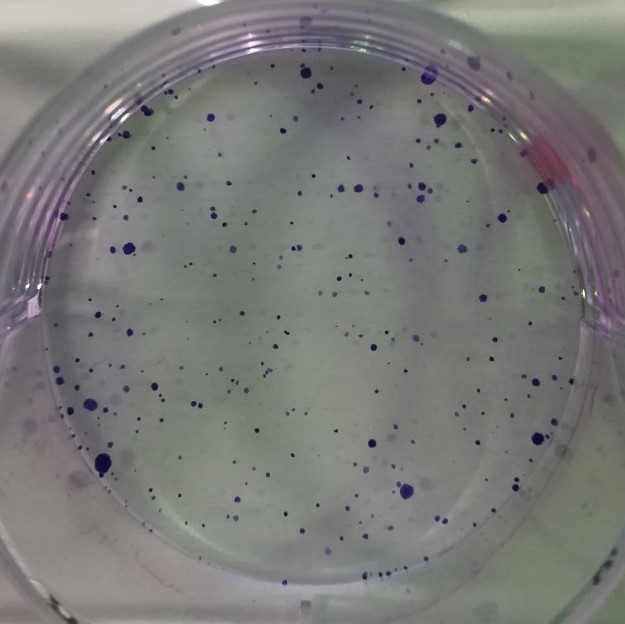

Supplement: Supplementary file 10 — Source data Fig. 4 [file 44320_2025_102_MOESM10_ESM.zip › Figure 4A/clonogenic_HT29_800c_Ctrl_Rep2.jpg]

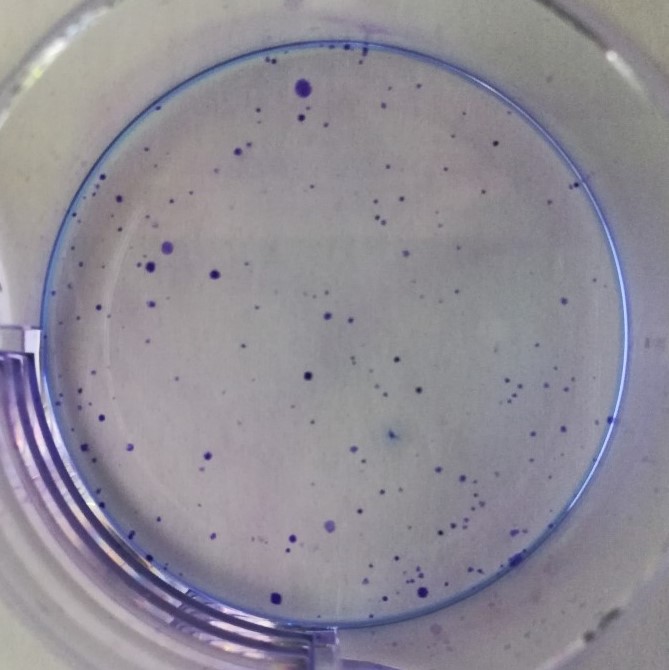

Supplement: Supplementary file 10 — Source data Fig. 4 [file 44320_2025_102_MOESM10_ESM.zip › Figure 4A/clonogenic_HT29_800c_KD_Rep3.jpg]

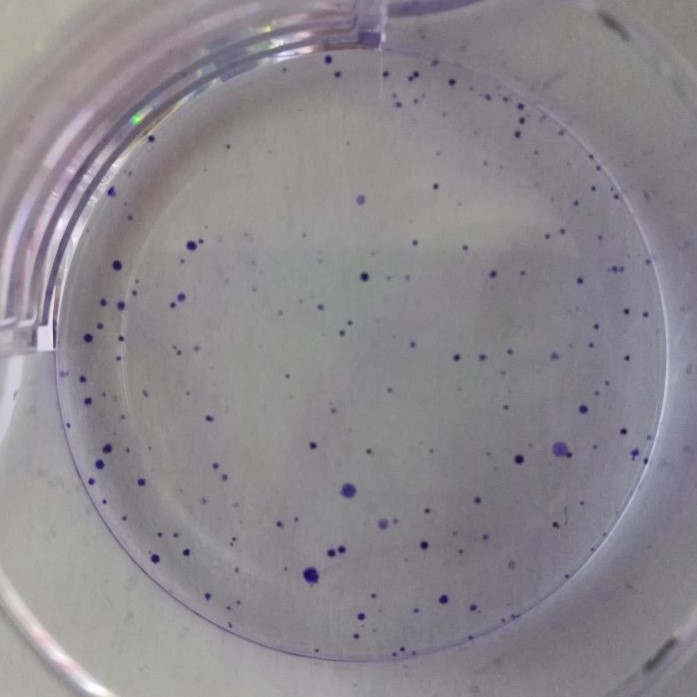

Supplement: Supplementary file 10 — Source data Fig. 4 [file 44320_2025_102_MOESM10_ESM.zip › Figure 4A/clonogenic_HT29_800c_KD_Rep1.jpg]

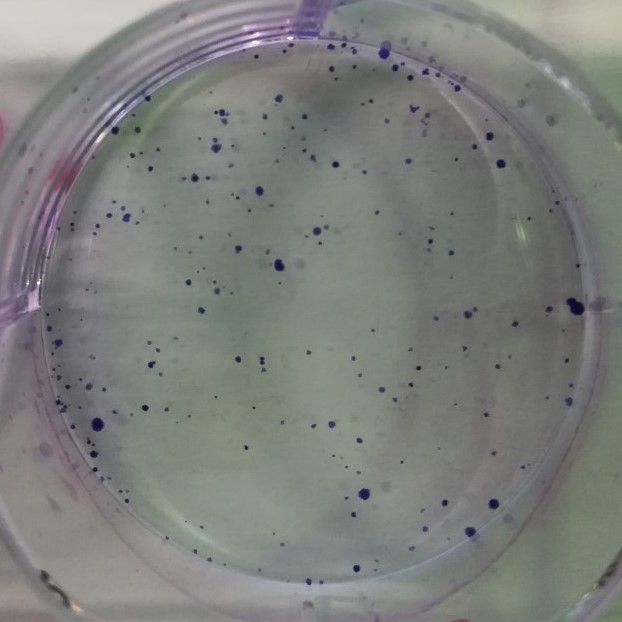

Supplement: Supplementary file 10 — Source data Fig. 4 [file 44320_2025_102_MOESM10_ESM.zip › Figure 4A/clonogenic_HT29_800c_Ctrl_Rep3.jpg]

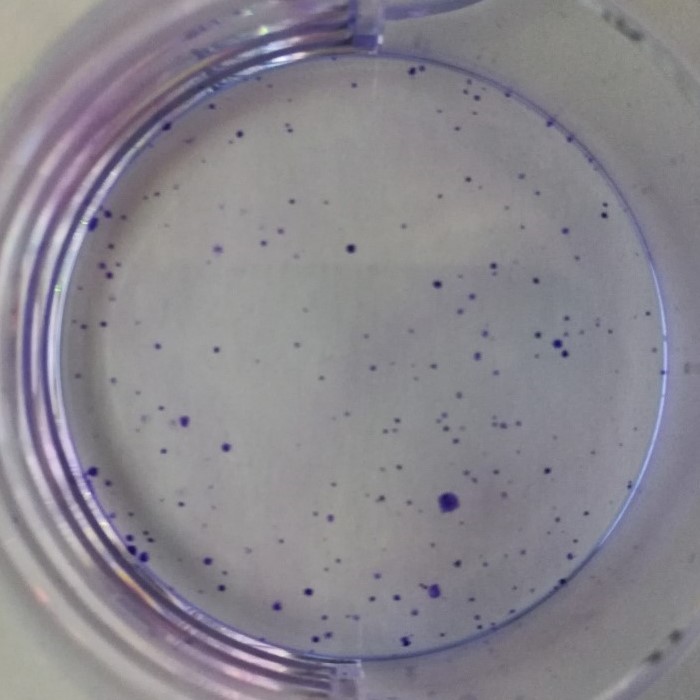

Supplement: Supplementary file 10 — Source data Fig. 4 [file 44320_2025_102_MOESM10_ESM.zip › Figure 4A/clonogenic_HT29_800c_KD_Rep2.jpg]

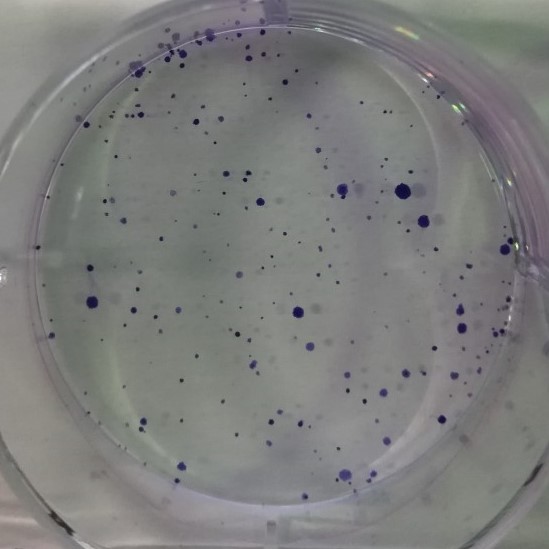

Supplement: Supplementary file 10 — Source data Fig. 4 [file 44320_2025_102_MOESM10_ESM.zip › Figure 4A/clonogenic_HT29_800c_Ctrl_Rep1.jpg]
