# Supplementary material for: Proteomics of colorectal tumors identifies the role of CAVIN1 in tumor relapse
Source: Mol Syst Biol. 2025 Apr 23;21(7):776–806. doi: 10.1038/s44320-025-00102-8 (PMC12222889; doi:10.1038/s44320-025-00102-8)
Supplement: Supplementary file 11 — Expanded View Figures [file 44320_2025_102_MOESM11_ESM.pdf]

Expanded View Figures

Figure EV1. Proteomics analysis pipeline.

(**A**) Frozen tumors were retrieved from the colorectal cancer biobank at the Department of Molecular Medicine, Aarhus University Hospital, Skejby, Denmark, processed using a cryotome for proteomics analysis. First three batches of samples were processed using in-solution digestion. Remaining batches were processed using Protein Aggregation Capture (PAC) protocol in the automatized King-Fisher platform. 10% of digested peptides was employed for two MS runs. Each sample was analyzed in duplicates in the Exploris480 coupled to EvosepOne using 60SPD pre-programmed gradient. MS runs were acquired in DIA mode using the FAIMS interface at CV- 45. MS datasets were acquired in three moments, batches 1 to 6 in October 2019, batches 7 and 8 in April 2020, and batch 9, comprising the 2nd cohort, was processed in July 2022. (**B**) Peptide Spectrum Matches (PSMs, in black) or peptides (in red) identified in quality control runs analysed before and in between the analysis of colorectal cancer protein batches. (**C**) Principal Component Analysis (PCA) showing MS run distribution based on protein intensity: (first row) using the output from Spectronaut, after data filtering for low quality runs, loess normalization and Left Censored Distribution imputation of missing values, (second row) after ComBat normalization to remove batch effect due to digestion procedure, (third row) after second ComBat normalization to remove batch effect due to sample batch proceeding. (**D**) PCA showing MS run distribution color coded by different clinical and demographic traits.

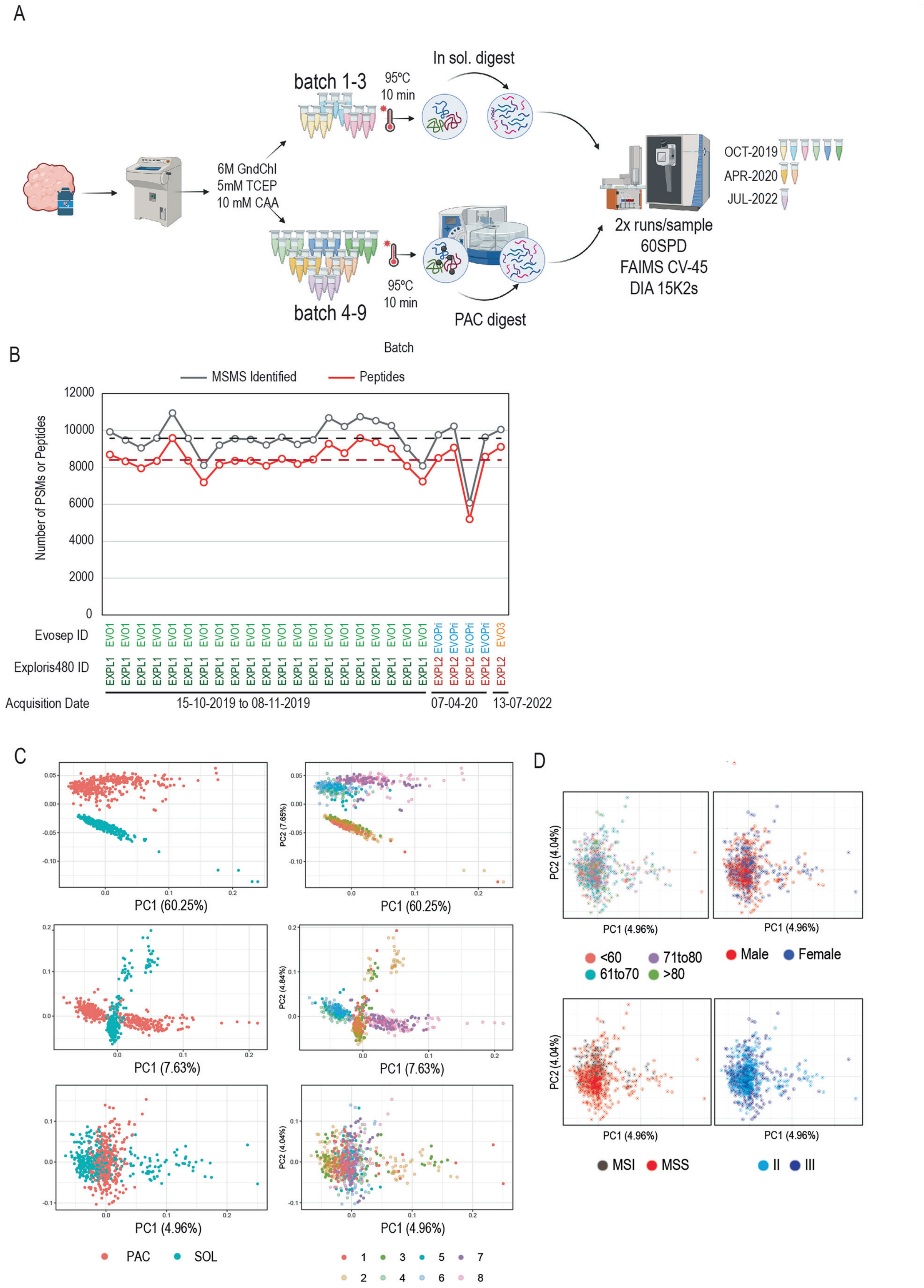
A
batch 1-3
95ºC
10 min
In sol. digest
6M GndChl
5mM TCEP
10 mM CAA
batch 4-9
95ºC
10 min
PAC digest
OCT-2019
APR-2020
JUL-2022
2x runs/sample
60SPD
FAIMS CV-45
DIA 15K2s
B
Batch
MSMS Identified
Peptides
Number of PSMs or Peptides
12000
10000
8000
6000
4000
2000
0
Evosep ID
EVO1
EVOPri
EVO3
Exploris480 ID
EXPL1
EXPL2
Acquisition Date
15-10-2019 to 08-11-2019
07-04-20
13-07-2022
C
PC1 (60.25%)
PC2 (7.65%)
PC1 (7.63%)
PC2 (4.84%)
PC1 (4.96%)
PC2 (4.04%)
PAC
SOL
1
2
3
4
5
6
7
8
D
PC2 (4.04%)
PC1 (4.96%)
<60
61to70
71to80
>80
Male
Female
MSI
MSS
II
III

 © The Author(s)

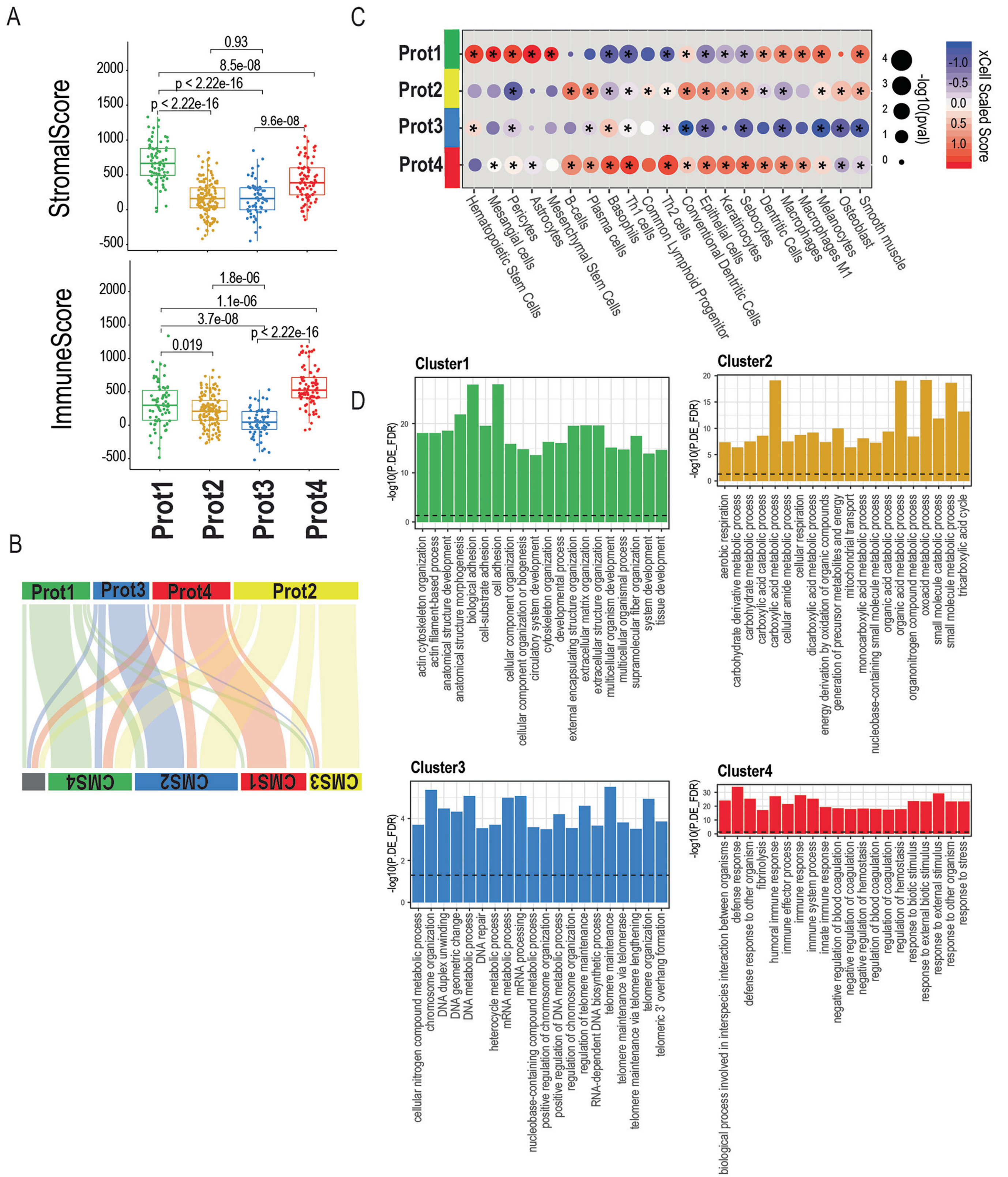
A
StromalScore
2000
1500
1000
500
0
-500
0.93
8.5e-08
p < 2.22e-16
p < 2.22e-16
9.6e-08
ImmuneScore
1.8e-06
1.1e-06
3.7e-08
p < 2.22e-16
0.019
Prot1
Prot2
Prot3
Prot4
B
Prot1
Prot3
Prot4
Prot2
CMS4
CMS2
CMS1
CMS3
C
Prot1
Prot2
Prot3
Prot4
Hematopoietic Stem Cells
Mesangial cells
Pericytes
Astrocytes
Mesenchymal Stem Cells
B-cells
Plasma cells
Basophils
Th1 cells
Common Lymphoid Progenitor
Th2 cells
Conventional Dendritic Cells
Epithelial cells
Keratinocytes
Sebocytes
Dendritic Cells
Macrophages
Macrophages M1
Melanocytes
Osteoblast
Smooth muscle
-log10(pval)
xCell Scaled Score
D
Cluster1
-log10(P.DE_FDR)
actin cytoskeleton organization
actin filament-based process
anatomical structure development
anatomical structure morphogenesis
biological adhesion
cell-substrate adhesion
cell adhesion
cellular component organization
cellular component organization or biogenesis
circulatory system development
cytoskeleton organization
developmental process
external encapsulating structure organization
extracellular matrix organization
extracellular structure organization
multicellular organism development
multicellular organismal process
supramolecular fiber organization
system development
tissue development
Cluster2
aerobic respiration
carbohydrate derivative metabolic process
carbohydrate metabolic process
carboxylic acid catabolic process
carboxylic acid metabolic process
cellular amide metabolic process
cellular respiration
dicarboxylic acid metabolic process
energy derivation by oxidation of organic compounds
generation of precursor metabolites and energy
mitochondrial transport
monocarboxylic acid metabolic process
nucleobase-containing small molecule metabolic process
organic acid catabolic process
organic acid metabolic process
organonitrogen compound metabolic process
oxoacid metabolic process
small molecule catabolic process
small molecule metabolic process
tricarboxylic acid cycle
Cluster3
cellular nitrogen compound metabolic process
chromosome organization
DNA duplex unwinding
DNA geometric change
DNA metabolic process
DNA repair
heterocycle metabolic process
mRNA metabolic process
mRNA processing
nucleobase-containing compound metabolic process
positive regulation of chromosome organization
positive regulation of DNA metabolic process
regulation of chromosome organization
regulation of telomere maintenance
RNA-dependent DNA biosynthetic process
telomere maintenance
telomere maintenance via telomerase
telomere maintenance via telomere lengthening
telomere organization
telomeric 3' overhang formation
Cluster4
biological process involved in interspecies interaction between organisms
defense response
defense response to other organism
fibrinolysis
humoral immune response
immune effector process
immune response
immune system process
innate immune response
negative regulation of blood coagulation
negative regulation of coagulation
negative regulation of hemostasis
regulation of blood coagulation
regulation of coagulation
regulation of hemostasis
response to biotic stimulus
response to external biotic stimulus
response to external stimulus
response to other organism
response to stress

© The Author(s)

Figure EV2. Differential regulation between proteomics subtypes.

(**A**) Boxplots of Stromal and Immune score distribution across proteomics subtypes. Different subtypes are compared using a two-sample t-test, and the *p*-value of the test is plotted on top. Due to limitation of the binary digits accuracy in R, when *p*-value is close to zero, it is reported as $p < 2.22e{-16}$. Subtype 1 $n = 74$, subtype 2 $n = 136$, subtype 3 $n = 61$, subtype 4 $n = 85$. (**B**) Sankey plot showing the correspondence between proteomics subtypes and RNASeq-based CMS subtypes. Boxplot limits indicate the 25th and 75th percentiles as determined by R software; whiskers extend 1.5 times the interquartile range from the 25th and 75th percentiles, outliers are represented by dots. (**C**) Cell type enrichment results from xCell, using RNASeq data as input and grouped by proteomics subtype. Statistical significance is represented as *p*-value, reported using the statistical test developed for this purpose and built inside xCell tool (Aran et al, 2017). (**D**) Gene Ontology Overrepresentation Analysis (fisher test, one side, FDR BH) for terms enriched in each cluster. Bars represent the −log10 FDR corrected *p*-value. Dashed line indicates FDR *p*-value of 0.05.

 © The Author(s)

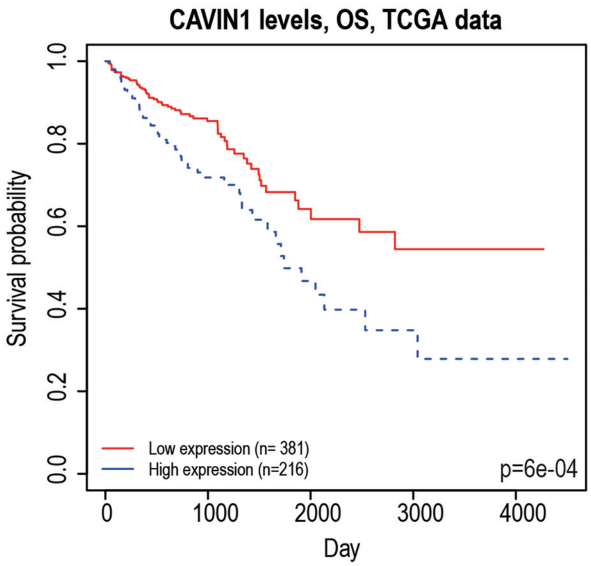
CAVIN1 levels, OS, TCGA data
Survival probability
1.0
0.8
0.6
0.4
0.2
0.0
Low expression (n= 381)
High expression (n=216)
p=6e-04
0
1000
2000
3000
4000
Day

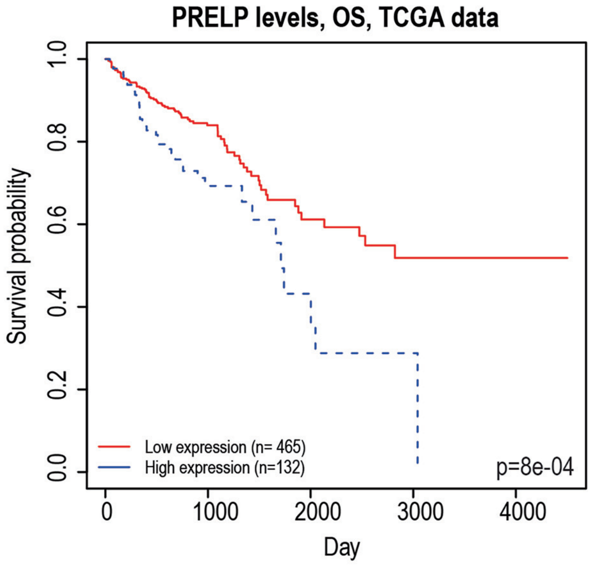
PRELP levels, OS, TCGA data
Survival probability
1.0
0.8
0.6
0.4
0.2
0.0
Low expression (n= 465)
High expression (n=132)
p=8e-04
0
1000
2000
3000
4000
Day

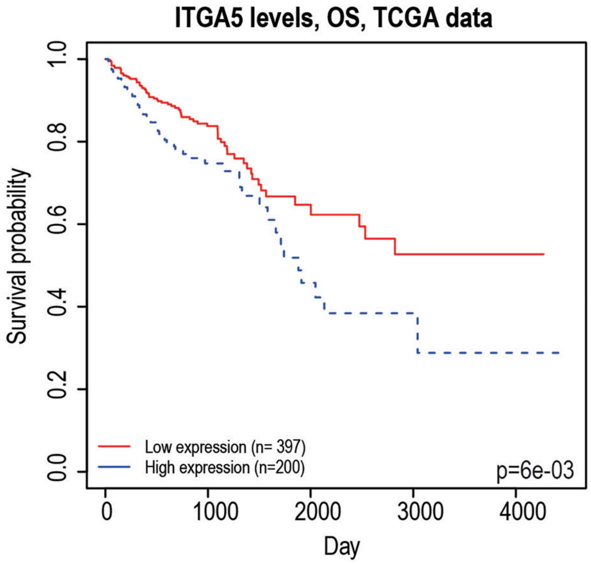
ITGA5 levels, OS, TCGA data
Survival probability
1.0
0.8
0.6
0.4
0.2
0.0
Low expression (n= 397)
High expression (n=200)
p=6e-03
0
1000
2000
3000
4000
Day

Figure EV3. Survival analysis of colorectal patients as a function of CAVIN1, PRELP and ITGA5 levels.

Kaplan–Meier plots summarizing results from analysis of correlation between mRNA expression level and patient survival. Patients were divided based on level of expression into one of the two groups “low” (<mean expression of PROTEIN) or “high”(>= mean expression of PROTEIN). X-axis shows time for survival (years) and the y-axis shows the probability of survival, where 1.0 corresponds to 100 percent. Data was obtained from The Human Protein Atlas (Uhlen et al, 2017).

© The Author(s)

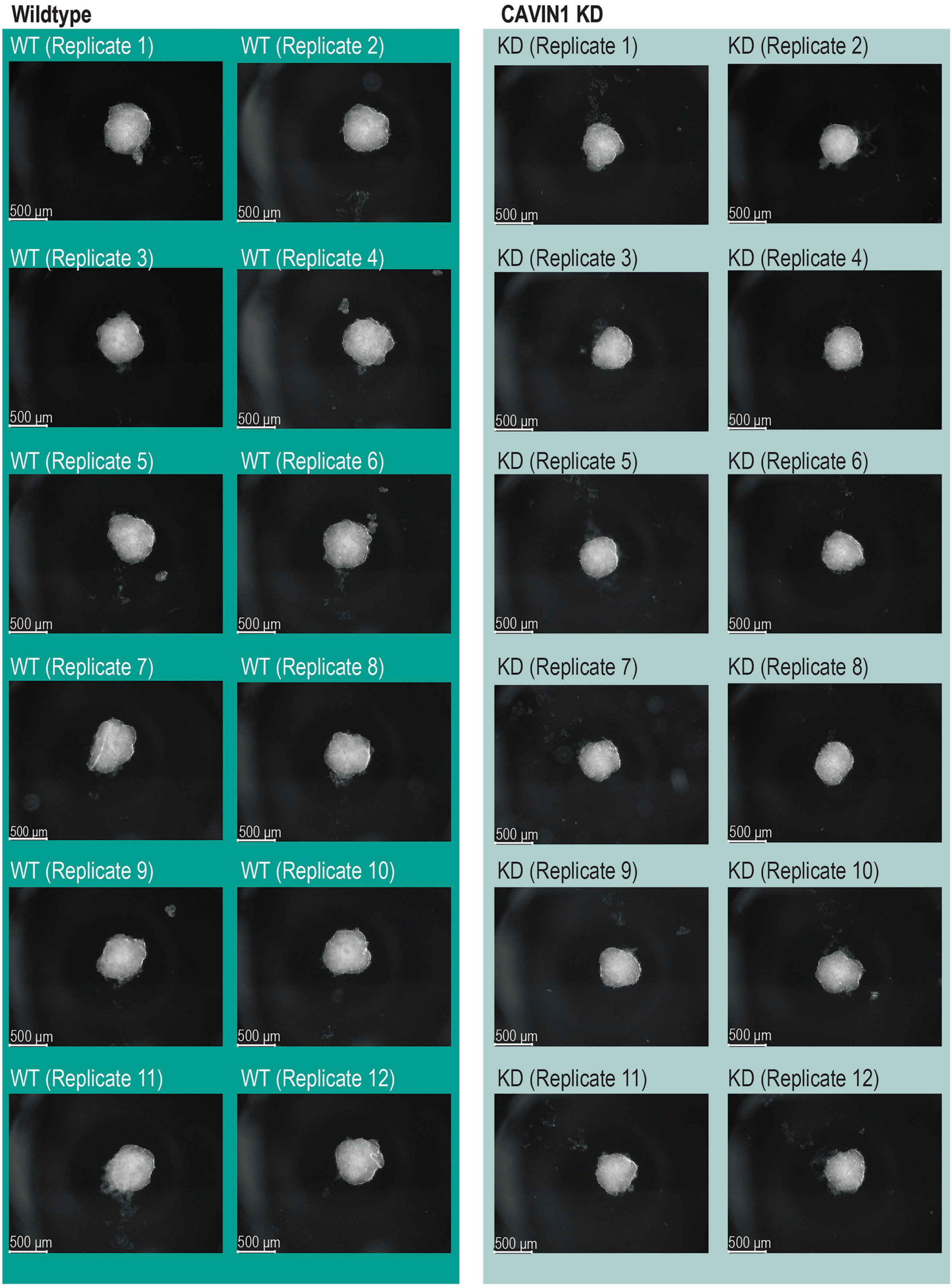
Wildtype
WT (Replicate 1)
500 μm
WT (Replicate 2)
500 μm
WT (Replicate 3)
500 μm
WT (Replicate 4)
500 μm
WT (Replicate 5)
500 μm
WT (Replicate 6)
500 μm
WT (Replicate 7)
500 μm
WT (Replicate 8)
500 μm
WT (Replicate 9)
500 μm
WT (Replicate 10)
500 μm
WT (Replicate 11)
500 μm
WT (Replicate 12)
500 μm
CAVIN1 KD
KD (Replicate 1)
500 μm
KD (Replicate 2)
500 μm
KD (Replicate 3)
500 μm
KD (Replicate 4)
500 μm
KD (Replicate 5)
500 μm
KD (Replicate 6)
500 μm
KD (Replicate 7)
500 μm
KD (Replicate 8)
500 μm
KD (Replicate 9)
500 μm
KD (Replicate 10)
500 μm
KD (Replicate 11)
500 μm
KD (Replicate 12)
500 μm

© The Author(s)

Figure EV4. Spheroid formation assay in WT and CAVIN1-KD HT29 cells.

Photographs of HT-29 derived spheroids, either wild type (left) or CAVIN1 knock down (right).

 © The Author(s)

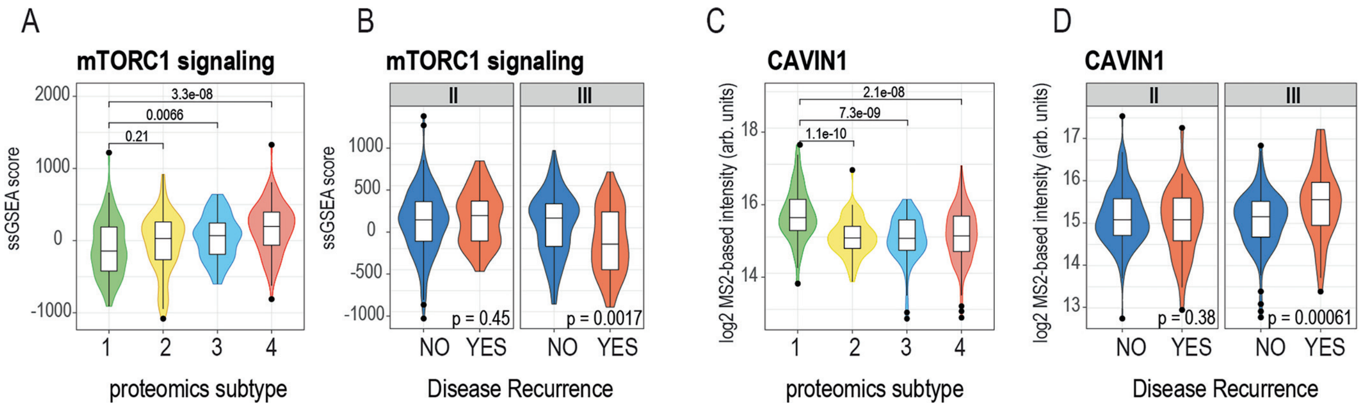

Figure EV5. Correlation between CAVIN1 levels and mTOR signaling with CRC proteomics subtypes.

(**A**) Boxplot of the ssGSEA score for Hallmarks gene set "mTORC1 signaling" in each proteomic subtype (using 1st and 2nd cohort samples) with the corresponding significance value from a two-sample t-test between subtype 1 and the others. (**B**) Boxplot of the ssGSEA score for Hallmarks gene set "mTORC1 signaling" (using 1st and 2nd cohort samples) grouped based on tumor stage (II or III) and disease relapse. *P*-values derived from a two-sample two-sided t-test statistical analysis. (**C**) Boxplot of CAVIN1 protein levels in each proteomic subtype (using 1st and 2nd cohort samples) with the corresponding significance value from a two-sample t-test between subtype 1 and the others. (**D**) Boxplot of CAVIN1 protein levels (using 1st and 2nd cohort samples) grouped based on tumor stage (II or III) and disease relapse. Statistical *p*-values derived from a two-sample two-sided t-test analysis. For (**A**) and (**C**): subtype 1 $n = 97$, subtype 2 $n = 112$, subtype 3 $n = 72$, subtype 4 $n = 125$. For (**B**) and (**D**): Stage II RFS Yes $n = 42$, RFS No $n = 177$; Stage III RFS Yes $n = 54$, RFS No $n = 133$. For all panels, boxplot limits indicate the 25th and 75th percentiles as determined by R software; whiskers extend 1.5 times the interquartile range from the 25th and 75th percentiles, outliers are represented by dots.

© The Author(s)
